# ‘This is just a little flu’: analysing medical populist discourses on the Covid-19 pandemic in Brazil

**DOI:** 10.1177/09520767221141121

**Published:** 2022-11-30

**Authors:** Erik Persson, Ewan Ferlie, Juan Baeza

**Affiliations:** 4616 King’s College London (KCL), UK

**Keywords:** Covid-19, pandemic, medical populism, public policy, health policymaking, critical discourse analysis

## Abstract

This paper explores the concept of medical populism to examine how Brazil has responded to the Covid-19 pandemic. Recognising the centrality of discourses in framing health policy, we employ Critical Discourse Analysis (CDA) to identify and analyse (1) what were the main discursive frames that characterised medical populism in Brazil’s Covid-19 crisis? and (2) how were these frames constructed, legitimated and reproduced in discursive meanings, structures and schemes of argumentation? Our study is an effort to inform the literature about medical populism and, more broadly, public health policymaking, administration and governance of health crises. Specifically, we seek to uncover the underlying discursive features of medical populism and expose how they frame public health policy. Our case study shows ample evidence that the main discursive frames underpinning medical populism during the Covid-19 crisis in Brazil reflected the most widely agreed attributes of populism as a strategic political discourse, notably an antagonistic depiction of the health problem, overpoliticisation and moral interpretation of political actors. However, our findings challenge some theoretical assumptions of extant conceptualisations of medical populism, thus providing greater insights into the concept of medical populism by demonstrating how this type of political discourse may incorporate different discursive meanings, structures and schemes of argumentation into its populist repertoire. This can help us anticipate patterns of action and narratives for preparing responses to future public health emergencies in an era of increasing post-truth politics, as populist discourses seem likely to influence public policy and governance for some time.

## Introduction

Unlike previous pandemics, the Covid-19 outbreak has been marked by huge mediatisation on news media and social media (Bal et al., 2020), allowing the instantaneous dissemination of a plethora of information, narratives and versions about the crisis and its developments. This favours the articulation of populist political discourses by charismatic leaders who often play a key role in spreading a rhetoric of fear, rumour and appeals to a division between “the people” and “an established power structure” (Speed and Mannion 2017; Canovan 1999; Kissas 2020), which directly impacts public policymaking and health strategies to fight the emergency.

The political nature of pandemic crises (Lasco and Curato 2019; Lasco 2020) makes them extremely susceptible to new populist discourses which, underpinned by post-truth politics, disdain for scientific expertise, economic insecurity and public fragmentation, aim to incite popular resentment against conventional political institutions to justify “alternative” health policies (Speed and Mannion 2017). In this context, while some political actors and policymakers try to address health crises through more, say, conventional technocratic, evidence-based approaches anchored in the expert knowledge of the medical professional establishment to reassure a frightened population, others resort to what Lasco and Curato (2019:1) define as “medical populism”, a political discourse “based on performances of public health crises that pit ‘the people’ against ‘the establishment’“. Put differently, medical populism is a type of strategic political discourse employed by populist leaders during health crises to overpoliticise, simplify and spectacularise complex public health problems (Lasco and Curato 2019; Lasco 2020).

In this paper, we explore the concept of medical populism as a theoretical heuristic device to examine how the Brazilian government responded to the Covid-19 pandemic during the first six months of the outbreak. Recognising the centrality of discourses in framing health policy (Bacchi 2000; Shaw 2010), we employ Critical Discourse Analysis (CDA) (Fairclough 2003) to identify and analyse (1) what were the main discursive frames that characterised medical populism in Brazil’s Covid-19 crisis? and (2) how were these frames constructed, legitimated and reproduced in discursive meanings, structures and schemes of argumentation? This will help understand how medical populism framed the Brazilian health policy for handling the crisis, given the extent of the epidemic in the country and governments’ difficulties in articulating a well-coordinated strategic response.

Considering that populist discourses usually revolve around charismatic leaders (Kissas 2020; Osuna 2021), we focus on the political strategy implemented by the far-right populist President Jair Bolsonaro and other key political actors at the highest levels of central and local governments in Brazil, as they played a decisive role in averting, or not, the worst outcomes of the crisis. We centre our analysis on the initial stages of the epidemic in Brazil, when the federal government was very powerful and dominated policymaking and Bolsonaro’s political discourse about the pandemic was in full force as a paradigmatic, ideal-typical case of medical populism that can offer relevant insights into its core defining attributes and logics of articulation in discourse (Laclau 2004).

Our study is an effort to inform the literature about medical populism and, more broadly, public health policymaking, administration and governance of health crises. Specifically, we seek to uncover the underlying discursive features of medical populism and expose how they frame political narratives, controversies and implications that can help explain the successes and failures of the strategy to fight the coronavirus emergency in an important Latin-American country, recognised by its comprehensive and universal public health system and relative expertise in dealing with past disease outbreaks. In so doing, our work adds to medical populism and health policy and administration research currents by illuminating how governments might respond to future health emergencies in an era of increasing post-truth politics (Speed and Mannion 2017).

### Medical populist discourse

The notion of populism has been increasingly employed in academic debates to understand changes in politics, public policy and administration in established liberal democracies (Speed and Mannion 2020). Indeed, new waves of right-wing and left-wing populist discourses have gained political weight across many countries since the 2008 economic downturn (Ramiro and Gomez 2017), with dissatisfied and anger voters turning against the “system” (e.g. the establishment, the elite, the globalisation, etc.) by supporting new political players or outsiders (Speed and Mannion 2017) who allegedly “voice popular grievances and opinions systematically ignored by governments, mainstream parties and the media” (Canovan 1999:2). For Lasco and Curato (2019:1), the concept of populism has become a “catch-all concept to diagnose the pathologies of political life”. This term, nonetheless, is highly politically contested and therefore still lacks a precise and agreed definition (Weyland 2001; Müller 2017; Moffitt and Tormey 2014; Osuna 2021). Populism can then be seen as a rhetorical strategy (Canovan 1999), a counter-revolutionary cultural backlash (Speed and Mannion 2017), a moralistic imagination of politics (Müller 2017), a performative (Kissas 2020) and thin-centred ideology (Mudde 2010), a political style (Moffitt and Tormey 2014) or logic (Laclau 2005). Each notion offers a promising theoretical-analytical lens to study populism depending on the object under investigation and focus of analysis.

To understand the Brazilian health policy response to Covid-19, we adopt the concept of populism as a strategic political discourse (Van Dijk 2006) “through which a personalistic leader seeks or exercises government power based on direct, unmediated, uninstitutionalized support from large numbers of mostly unorganized followers” (Weyland 2001:14). Such a political discourse involves an “appeal to ‘the people’ against both the established structure of power and the dominant ideas and values of the society” (Canovan 1999:3), including reactions against expert opinion-formers of the academy and professionalised civil service. As exemplified by politicians like Jair Bolsonaro, Donald Trump, Viktor Orbán and Rodrigo Duterte, populist leaders often pursue a policy agenda that eschews established institutional structures of checks and balances and by-passes policy experts and professional public managers (Speed and Mannion 2017). In contexts of health-related crises, a populist policy agenda may result in increasing politicisation of important medical or technical aspects of the health emergency, which may lead to poorly designed and implemented health policy responses. Politicisation, or the extent to which political orientations influence public trust in science and institutions and their organisation, is a central element in the notion of medical populism, a political discourse “that constructs antagonistic relations between ‘the people’ whose lives have been at risk by ‘the establishment’” (Lasco and Curato 2019:1). Simply put, medical populism refers to how populist political actors exercise power in public policymaking while discursively framing and devising solutions to health crises (Speed and Mannion 2020). Such a political discourse can be characterised by three basic attributes:

**- an appeal to “the people”**, through which populist leaders build on threats to public’s health and safety to create a shared imaginary of “the people” as aggrieved parties;

- **performance of health issues as a crisis**, through spectacular, dramatised or distorted representations of the problem in order to make a case for immediate responses; and

**- simplified discourse**, through communicative practices that draw on explicit anti-intellectualism to offer common-sense solutions to complex problems (Lasco and Curato 2019; Lasco 2020).

All those three elements are inscribed in the need to forge divisions between the ingroup from the outgroup. Furthermore, implicit in them is a distrust of evidence-based policy approaches and the depreciation of experts and professional bureaucrats (Speed and Mannion 2020). Taking shape in discursive practices, medical populism also implies a performative power in the framing of the health issue, in that in public policy and administration influential political and social constructions are produced and negotiated through discourses (Fischer 2003), which help explain important socio-political dynamics and variations that dictate policymaking.

## Methodology

This work is based on Critical Discourse Analysis (CDA) (Fairclough 2003), which enables a critical approach to political discourses (Van Dijk 2003, 2006) through “a perspective which focuses on the reproduction and contestation of political power through political discourse” (Fairclough and Fairclough 2012:17). As Wodak and Meyer (2009:7) explain, CDA aims to provide “a proper understanding of how language functions in constituting and transmitting knowledge, in organizing social institutions or in exercising power”. We adopted CDA to explore what the discursive features of medical populism look like and how they are articulated by political actors to reach or exercise power in health crises. CDA, in this case, is a fruitful methodological approach to delve deeper into the underlying discursive meanings and structures of medical populist claims, thus helping clarify how these integrate a wider ideational or discursive repertoire (Osuna 2021) of political discourse populist leaders draw from to influence health policy processes.

Considering that texts “are the relevant units of language in communication” (Wodak 2001:6) and “probably the most fully studied form of discourse within CDA” (Scollon, 2001:175), we applied a textually oriented discourse analysis (Fairclough 2003). To address the matter of representativeness of the texts selected (Widdowson 1995) and avoid a “go fishing” approach to arrive at data with no precise plan (Titscher et al., 2000), we followed Reisigl and Wodak’s (2009) criteria for a systematic collection of text material:

**- specific political units**: since our study aimed to identify and examine medical populist discourses about the handling of the Covid-19 crisis in Brazil, the political units were defined to encompass health bodies and institutions of the executive and legislative powers at the federal level of government and, to a lesser extent, relevant state and municipal authorities. When appropriate, we also included texts from international organisations and authorities (e.g. WHO) in order to compare domestic and international policies, interpret political stances and narratives, or provide context (see Appendix A).

**- specific fields of political action and policy**: texts should be centred on health policy and political discourses about the corona crisis in Brazil, thus comprising important discursive events relating to policy decision-making and implementation (e.g. law making-procedures, public control and administration of the Covid-19 emergency), health governance and policy coordination, and formation of public attitudes, opinions and will (e.g. political advertising, media coverage).

**- specific social, political and scientific actors**: taken as prominent and exceptional voices of far-right discourses on Covid-19 (Wondreys and Mudde 2020), the focus was given to President Jair Bolsonaro, his ministers of state and close associates, as well as governors, mayors, politicians and other health actors with national projection or influence in relation to our topic of interest.

**- specific discourses**: we selected political statements, declarations and speeches about the coronavirus pandemic, as well as texts about the overall strategic response by central and local governments.

**- specific period of time**: the timeframe selected for the data collection was January–June 2020. The first six months of the outbreak in Brazil clearly reflected how Bolsonaro’s government reacted to a novel crisis and tried to incorporate it into a wider far-right repertoire of political discourse and goals. At this stage of the pandemic the federal government was very powerful, favouring the ascendence of medical populism and its major impact over health policy; subsequent phases became more mixed due to some policy learning from other countries, intensification of internal criticism and opposing interventions that put heavy weight on Bolsonaro’s administration and populist approach. Therefore, both the selected case and specified time interval have helped build up a coherent and comprehensive picture of how medical populism is mobilised through discursive practices that can become highly influential, or even dominate political debates and policymaking, in the early stages of a health crisis.

**- specific semiotic media and genres**: while our sample was purposely selected, we tried to include a variety of non-reactive data (Meyer 2001) to ensure reliability, relevance and triangulation, thus helping us disentangle discourses through analysing different ways of speaking about our core topic (Shaw 2010). Our sample, therefore, comprises publicly available texts about Covid-19-related policymaking extracted from multiple discursive genres, such as TV and radio interviews, newspapers, public speeches and declarations, press conferences, government reports and guidelines, official communication, institutional websites, laws, regulations and other legal documents, and particular Twitter posts and YouTube videos (see Appendix A).

Following the aforementioned criteria, we used a simple Google search to locate key documents and speeches of political actors involved in the corona crisis policymaking. Query terms such as “Covid-19”, “*coronavírus*”, “*pandemia*”, “*epidemia*” and other related terms were employed to identify and access relevant national sites, online news and documents. During this process of data gathering, we discarded any irrelevant information by reviewing and retaining only statements and extracts that met the inclusion criteria. Although only materials produced in Portuguese were selected, we reviewed international news coverage on a few occasions to compare information, double-check translations or consult original sources. Also, quoted or referenced subjects and documents were added to the data in order to allow the analysis of intertextuality (e.g. differences between direct and indirect reporting) (Fairclough 2003). Whenever possible, we watched videos of interviews, speeches and declarations to collate spoken and written data.

The systematisation and organisation of data began with the transcription of all interviews, speeches, press releases, videos, and compilation of other documents. Texts were then copied and pasted into files in Microsoft Word format to compose a discursive corpus with a total of 927 excerpts organised in a daily sequence of discursive events. The discursive corpus was then entered in the software package NVivo11, which facilitated coding, text searches, counting, and intersections of codes.

The data analysis approach used in this study was based on a combination of inductive and deductive research logics, meaning that our analysis and theorisation processes were iterative, moving between data and theory to explain the findings. The analysis started with open coding through close line-by-line reading of the raw data (discursive corpus). To each excerpt of text a code was assigned to label a concept or phenomenon of interest. A single excerpt of text was coded with multiple codes when different phenomena were found in it. Coded text length ranged from one sentence to multiple, long excerpts. Stage two coding consisted of axial coding, with more focused textual analysis to generate 1^st^ order concepts using CDA to explore discursive properties underlying medical populist narratives present in the actual words of informants and documentary material. We then used theoretical constructs of populism (Osuna 2021; Van Dijk 2006; Weyland 2001) and medical populism (Lasco and Curato 2019; Lasco 2020) to reanalyse initial concepts and look for patterns and relationships between them in order to refine data and distil researcher-centric 2^nd^ order themes that could describe the main discursive frames characterising medical populism in the Brazilian health policy for handling the corona crisis. The next stage of analysis involved applying policy-as-discourse framing to distil themes further into aggregate frames to offer new theoretical insights into how medical populism are embedded in broader political discourses mobilised by populist leaders to influence policy processes. Table 1 provides an overview of our findings.

**Table 1.** Data overview.

Aggregated frames	2^nd^ order themes	1^st^ order concepts	Discursive examples
**Antagonistic depiction of the pandemic crisis**	**Minimising the pandemic**	Lexicalisation; word choices and euphemisms to evaluate the pandemic; recontextualisation	41. It’s **a flu**, but it’s **a flu** that you’re going to overcome. [Minister of health 1/press conference/Feb/2020] 127. We obviously have a crisis at this moment, **a little crisis**. [President/newspaper/Mar/2020] 881. In my case, with my history as an athlete, if I were infected with the virus, I would have no reason to worry. I Would feel nothing, or it would be at most just a **little flu** or a **bit of a cold**. [President/TV speech/Mar/2020]
		Presupposition of Covid-19 as an elderly disease	164. What we must do is to spend resources properly, to make good use of them. This is possible only if the community, the society protects our **elderly** and **people with comorbidities**. [Minister of health 1/press conference/Mar/2020]
		Naturalisation of Covid-19-related deaths as unavoidable; manipulation; focusing on older people to preserve the economy	224. **Disabled or older people** who obviously have disabilities due to their age can die, because any flu can lead to death. [President/newspaper/Mar/2020] 540. […] It’s like a war. Unfortunately, **we lose soldiers in a war**, and they are human beings. Here, **we’re going to lose lives too** in this war against the virus. [President/newspaper/Apr/2020] 474. I’m sorry, some people will die, they will die, that’s life. **You can’t stop a car factory because of traffic deaths**. [President/newspaper/Mar/2020]
	**Redramatising the pandemic**	Hyperbole; exaggeration of media coverage	239. We were expected to face this [Covid-19]; however, such hysteria is wrong, as if it were **the end of the world**. [President/newspaper/Mar/2020]
		Reverse panicking narrative; press hysteria; mainstream media spreading dreadful and biased news	127. […] this coronavirus issue is much more of a fantasy. It’s not all that **the mainstream media spreads or propagates** worldwide. [President/newspaper/Mar/2020] 46. We have to keep calm and spread calm to avoid **scaremongering** […]. [Minister of health 1/press conference/Feb/2020] 245. […]. We should not lead this to turn into **hysteria and desperation**! [minister of health 1/Twitter/Mar/2020]
		Number game rhetoric	364. I See the numbers reported [by the ministry of health] in those projections. **I Find them exaggerated**. [President/newspaper/Mar/2020] 458. If everybody is dying due to coronavirus so this signals that **the state is faking people’s *causa mortis***. We cannot accept the political use of numbers. [President/newspaper/Mar/2020] 459. [Death toll] It’s too high in São Paulo […]. **I Do not believe in those numbers**, especially since that decree was enacted by the governor. [President/newspaper/Mar/2020]
	**Appealing to a false dilemma**	Argument that restrictive measures were wrecking the economy; interlocking goal-achievement and problem-solution patterns	220. There will be a **much bigger [economic] chaos** than this virus can cause in Brazil. […]. [President/newspaper/Mar/2020] 272. […] there’s no point in locking everything down and lacking food that is ready to be delivered, because then **you address one thing but run out of other**. [Minister of health 1/press conference/Mar/2020]
**Overpoliticisation**	**Undermining conventional approaches**	Populist response opposing traditional, technocratic responses	481. What **people ask me** most is to get back to work. [President/newspaper/Mar/2020] 605. […] the federal government has been adopting irresponsible actions that **oppose health protocols that have been approved by the scientific community** and applied by other heads of state worldwide. [CFOAB, Lawsuit against Noncompliance with Fundamental Precept/ADPF n. 672/2020/Apr/2020] 537. […] I do not intend to dismiss him [minister of health 1] in the middle of the war, I don’t. However, **he is a person who has overreacted at some point**. He knows there’s a hierarchy between us. [President/YouTube channel/Apr/2020] 687. He [the President] clearly expresses that **he wants another stance for the ministry of health** […]. [Minister of health 1/press conference/Apr/2020]
	**Underrating drastic measures**	Hortatory discourse; argument that lockdown was necessary for at risk groups only	511. The side effects of measures to combat coronavirus cannot be **worse than the disease itself**. [President/TV speech/Mar/2020] 431. […] therefore, **we have to protect those [older] people and those who are in risk groups** by giving them all care, affection and respect. **For them, quarantine**. For the others, social distancing, extra attention and a great deal of responsibility. [#BrazilCannotStop ad campaign/Mar/2020]
	**Prioritising the economy**	Problem-solution pattern; Covid-19 crisis conceived of as an economic problem	213. **There is** indeed a danger, but this issue **is being oversized**. The economy can’t stop for coronavirus. [President/newspaper/Mar/2020]
		Opportunity-taking pattern; taking the economic reform agenda forward	122. […] **we need economic reforms now**. […] **[economic] reforms are the best response to the crisis**. We’ll be dispatching the **administrative reform**; the **federative pact** is already there [in the Parliament]; we’ll be dispatching the **tax reform**, and we’re going to move forward with the work. [Minister of economy/press conference/Mar/2020]
		Legitimation; calling for institutional authority; direct and indirect reporting	513. Have you guys seen what the WHO Director-general has said? Has anyone seen that? He basically has said, especially concerning informal workers: “**they gotta work**”. [President/newspaper/Mar/2020]
	**Claiming simplistic solutions**	Relational processes; identifying vertical isolation approach and (hydroxy) chloroquine as effective solutions; attributing value to alternative solutions	469. […] [hydroxychloroquine] is being used in several hospitals. […] **the cure is there**. [President/newspaper/Mar/2020] 668. […] the population is not debating whether there will be beds if they get sick, whether there will be tests. No, **they are talking about chloroquine**, a drug for which proofs of safety and effectiveness are still lacking. [Director of the department of Immunization and Transmissible diseases of the ministry of health/newspaper/Apr/2020]
**Moral interpretation of political actors**	**Drawing on normative polarisation**	US vs THEM dichotomisation; logic of equivalence; legitimation through a normative framework	652. President’s **calls for people to leave isolation make the virus stronger** and [the city of] Manaus weaker. President Bolsonaro is the main ally of the virus today. [Mayor of Manaus/newspaper/Apr/2020] 217. We’re not going to overestimate this issue. **We cannot let some authorities start imposing a ban on this or that**. [President/newspaper/Mar/2020]
		Positive self-presentation; emphasising ingroups’ good actions; minimising ingroups’ bad actions	877. The President **has provided the health area with all conditions** to strengthen the SUS […]. **Support has been given** to governors and mayors. [Minister of Citizenship/newspaper/May/2020]
		Negative other-presentation; minimising the other’s good actions; accentuating other’s bad actions	825. **I’m not fiddling with anything**. Health is life. The decision to close businesses is up to the respective governor or mayor. **I’ve been doing what is my responsibility**. [President/newspaper/May/2020] 192. We have to look at the extent to which these **measures [taken by governors]** will affect our economy. [President/newspaper/Mar/2020]
		Blame avoidance; keeping away from blame-generating events Blame allocation; shifting blame to other actors or entities	345. I Am concerned about people’s lives and **unemployment caused by these negligent governors**. [President/newspaper/Mar/2020] 654. We had more than 70% compliance with horizontal isolation. People were staying at home. There was a huge calm. **When the President opposed this**, people started to find some logic in his position, and **many got back to the streets**. The compliance dropped to slightly over 50%. Today people are insistently outside their homes. [Mayor of Manaus/newspaper/Apr/2020]
	**Adopting a confrontational approach**	Controversies leading to poor coordination among central-local governments	879. Our lives have to go on. Jobs must be kept. **We must get back to normal**. [President/newspaper/May/2020] 820. The Decree **recommends mayors** of the state [of Rio de Janeiro] **adopting some form of ‘lockdown’ as a measure of social isolation** aimed to curb the spread of the disease. [Governor of Rio de Janeiro/newspaper/May/2020] 447. I Am appalled by the President’s speech on isolation measures, prudent measures that have been taken by several state governors and mayors. I’ve come to inform the people of Santa Catarina that on Wednesday, March 25, **we will be initiating a seven-day quarantine** as determined by Decree, seven days to stay at home, which is the safest place. [Governor of Santa Catarina/newspaper/Mar/2020]
		Contradiction and ambiguity resulting in poor coordination at the central level	605. […] the [federal] government has **not always been making adequate use of its prerogatives** to address the public health emergency but constantly acting in an insufficient and precarious way. [CFOAB, Lawsuit against Noncompliance with Fundamental Precept/ADPF n. 672/2020/Apr/2020] 713. **Differing discourses create differentiated individual attitudes**. […] **many people started to believe that isolation is not effective**, and they are now going to the streets, increasing the counting rate. [Director of the department of Immunization and Transmissible diseases of the ministry of health/newspaper/Apr/2020]

Source: Authors.

### Contextualising the Covid-19 crisis in Brazil

Brazil is a large upper-middle income country currently confronting economic stagnation, rising public debt, severe social inequalities and political polarisation. This scenario has been aggravated by the new coronavirus epidemic, which puts pressure on the economy and causes a heavy strain on the Unified Health System (SUS) (Almeida 2021), the country’s main weapon to fight the health crisis. Despite persisting underfunding, inequalities in access to services and management challenges (Massuda et al., 2018), the SUS continued to function during the outbreak, mostly because of contingency actions adopted by local governments and health institutions in view of the vacuum of political leadership and weak coordination at federal level (Barberia and Gómez 2020).

When the Covid-19 pandemic started to spread globally by January 2020, Brazil counted on an effective national Department of Epidemiological Surveillance (SVS) that supported the Ministry of Health (MH), states and municipal health authorities in elaborating initial contingency plans through analysis of suspected cases and publication of epidemiological bulletins. Based on technical and scientific data available, the MH declared a public health emergency of national concern on February 6. This demarcated Stage One of the Brazilian health policy response – **Gathering information and planning contingency actions**.

The MH constituted the central body and lead ministry elaborating and managing the country’s strategy. Working closely with the WHO for assessing the outbreak and sharing scientific information, the technical branch of the MH provided public health expertise, advice, resources and strategic oversight to support regional responders. With the expansion of the crisis, other areas of government were included in the containment strategy, comprising several ministries and subordinate agencies at arm’s length from the MH. In addition, a special government cabinet for crisis management was instituted under the Presidency of the Republic.

Responding to the high rate of community transmission, most regional authorities determined extensive non-pharmaceutical interventions (e.g. stay-at-home advice, school closures, restrictions to public transport, limits on social gathering) to slow the spread of coronavirus. These measures characterised Stage Two – **Restraining the outbreak**. However, since the beginning of the outbreak, President Bolsonaro underrated the pandemic. His commitment to the economic agenda remained unwavering (Barberia and Gómez 2020) even when growing cases and the death toll led to a clamour from health experts and opposition leaders for tougher lockdown type measures (Lasco 2020).

Aiming at re-election in 2022, Bolsonaro sought to maintain support from the economic sector by urging people to keep to their routines and continually ignored scientific advice on social distancing and mask use. Under these circumstances, measures advocated by the MH remained short in comparison to other nation’s implementation of mandatory lockdowns, widespread testing and more rigorous precautionary actions (Mellan et al., 2020; Kim 2021). Bolsonaro administration’s narrow-minded approach influenced health authorities to move on to Stage Three – **Focusing on patient care**, with the intensification in the treatment of infected people presenting mild symptoms to prevent more critical conditions. Health authorities then adopted an approach to test only individuals with severe symptoms, the elderly, health professionals and other key workers, hence dismissing the other nations’ strategy of widespread testing, which led to difficulties in assessing the growth of the epidemic in Brazil.

Although previous presidents have also appointed health ministers and senior officials to forge political compromise and coalitions (Barberia and Gómez 2020), the influence of Bolsonaro’s populism on the MH reveals more blatant efforts of direct politicisation (Peters 2013) to have political loyalists, or at least sympathisers, replacing career officials in leading positions within this important national health body. President Bolsonaro, his family and associates have also been strongly criticised for disseminating disinformation and fake news during the pandemic and promoting the expanded use of chloroquine/hydroxychloroquine despite limited scientific evidence about their effectiveness.

Bolsonaro’s dismissive stance towards the Covid-19 crisis exacerbated bitter divisions in the Brazilian political environment (Lasco, 2020). His radical right-wing populist politics is exerted through polarisation, authoritarian leadership, appeals to conservative family values, Judeo-Christian morals and a strong economy (Barberia and Gómez 2020), as well as massive use of social media to mobilise followers and exercise political power.

Nonetheless, without a majority in Congress and facing opposition efforts to opening impeachment proceedings amidst declining popular general approval (his followers comprise about 25% of the population), Bolsonaro had to resort to bargaining political support from centre-right parties, which has weakened his populist discourse against the “established structure of power”. Also, Bolsonaro administration’s attempts to abandon restrictions and resume social functioning were limited by judicial decisions, which consistently upheld the legality of measures imposed by local authorities (Barberia and Gómez 2020).

Even so, data show that the federal and local governments acted in an uncoordinated way in implementing Stage Four – **Relaxing restrictions and resuming social functioning**, despite the severity of the outbreak in all regions, lack of ICU beds, significant under-notification of confirmed cases and increasing death toll. As a result, during the first six months of the pandemic, Covid-19 grown quickly in Brazil, which became an epicentre of coronavirus in Latin America (Mellan et al., 2020; Almeida 2021). As of August 2022, Brazil had reported 34 million cases and over 679,000 deaths. These, probably underestimated figures, ranked Brazil among the most affected nations in terms of total Covid-19 cases and deaths.

### Discursive framing of the Brazilian health policy for Covid-19

In our first research question, we sought to understand the main discursive frames that characterised medical populism in Brazil’s Covid-19 crisis. To achieve this, we adopted a policy-as-discourse approach combining framing and discourse analysis. Frames are symbolic or heuristic interpretive constructs which organise meanings and concepts through which we order experiences in and responses to social reality (Triandafyllidou and Fotiou 1998; Garvin and Eyles 2001), thus shaping our evaluation and understanding of the environment around us (Koon et al. 2016). The policy-as-discourse framing approach is based on a deep reflection on the social and political contours of a particular policy discussion (Shaw 2010). It seeks to address a certain policy agenda to understand how discourses shape policy problems, that is, how policy is constructed and represented in terms of what can be discursively thought about and acted upon as possible or impossible, as dispensable or necessary, as desirable or undesirable (Bacchi 2000). Hence, the policy-as-discourse framing perspective was a fruitful analytical device to explore the essentially discursive nature of medical populism, a type of political discourse that involves a process of deliberation in which power relations play an integral role (Shaw 2010) and every choice of wording always has a political meaning (Fairclough and Fairclough 2012).

Our case study shows ample evidence that the main discursive frames of medical populism reflected some core ideational, discursive and performative attributes of populism as a strategic political discourse (Osuna 2021; Van Dijk 2006). We present three of those frames here: (1) **an antagonistic depiction of the crisis**, through minimising, redramatising the pandemic and appealing to a false dilemma; (2) **overpoliticisation**, through undermining conventional approaches, underrating drastic measures, prioritising the economy and claiming simplistic solutions, and (3) **moral interpretation of political actors**, by drawing on ideological polarisation and adopting a confrontational approach. To address our second research question, we used CDA to demonstrate how those frames were discursively constructed, legitimated and reproduced, thus uncovering the inner logic of medical populist discourses (see Table 1).

### Antagonistic depiction of the pandemic crisis

**Minimising the pandemic**: whilst the literature on medical populism suggests populist leaders usually build on moral panics (Cohen 2011) to exaggerate threats to public health, our data show that medical populist discourses (Lasco and Curato 2019; Lasco 2020) on Covid-19 health policy in Brazil have clearly expressed a discursive frame of downplaying the seriousness of the pandemic. President Bolsonaro, for example, repeatedly represented Covid-19 as a “flu” to situate the novel coronavirus infection as a common health problem. In this medical populist discourse, the lexicalisation or wording (Fairclough 2003) of the Covid-19 disease as a “little flu”, “a bit of cold”, “elderly disease”, “hysteria”, “psychosis” or “fantasy” reflects the rhetorical figure of euphemism (Van Dijk 2006) through using mitigating adjectives to evaluate the epidemic and avoid or reduce other potential negative meanings that could denote or reinforce the severity of the crisis and, consequently, legitimise more draconian contingent measures. Such wording evoked particular ways of recontextualising the epidemic in Brazil and framing the federal government’s selection of information, priorities and more reactive strategies to control the outbreak. The medical populist depiction of Covid-19 also involved the presupposition that elderly, ill or disabled people constituted the main group potentially affected by the outbreak. Similarly, the naturalisation of death due to Covid-19 infection was present in Bolsonaro’s public statements in which the allegedly unavoidable number of deaths became gradually represented as self-evident, while official statistics showed a rising death toll. Within policymaking, presuppositions are strategic linguistic devices (Fairclough 2003) for framing policy issues, often employed to naturalise the truth of certain assumptions, even if such truth is not established at all (Van Dijk 2006). They are effective instruments of manipulation in populist discourses because they are very difficult to contest (Fairclough 2003), often inferable from general knowledge (Van Dijk 2006) and more or less pervasively held throughout social domains and organisations (Fairclough 2003). Hence, Bolsonaro administration framed Covid-19 deaths as an “older people issue” to orient the health policy response towards the care of this specific group and justify government actions aimed at preserving the functioning of the economic sector.

**Redramatising the pandemic**: according to the literature, populist leaders tend to call upon dramatisation of the health problem in order to justify immediate and extreme actions (Lasco and Curato 2019; Lasco 2020). Our case study illustrates an intriguing counterintuitive example of medical populist discourse that not only questioned the spectacularisation of the crisis to avoid drastic measures but also incited public reaction against a presumed overdramatisation of the pandemic. Such a narrative can be seen as an attempt to “redramatise” the crisis to accommodate it to a broader repertoire of political discourse against the mainstream media identified with the interest of “the system”. For example, Bolsonaro and his ministers claimed that the Covid-19 crisis was being vastly oversized in Brazil, notably by the traditional media; they resorted to a struggle over meaning (Fairclough 2003) with the media coverage, arguing that its biased and exaggerated reporting of the pandemic was nothing but “a trick” to spread panic and ruin his popularity. Redramatisation was associated with Bolsonaro’s narrative of conspiracy (Lasco 2020), according to which “*much of the mass media were acting against*” his government to “*spread a feeling of dread*”, which created “*a perfect scenario potentiated by the media for real hysteria to get disseminated across the country*” [383./TV speech/Mar/2020]. The Minister of Health 1, Luis Henrique Mandetta, also fuelled the redramatisation narrative by endorsing a negative presentation (Van Dijk 2006) of how important news outlets were framing and covering the epidemic in Brazil. In his words, “*the greed for outselling front pages is part of the outbreak* [reporting]*, for the more dramatic a front page is, the higher the number of visits it gets* [online] *is*” [273./press conference/Mar/2020]. Furthermore, with Covid-19 being the first global pandemic in times of social media (Bal et al., 2020), panicking around Covid-19 was conceived as natural and “*inflated by the internet*” because of massive flows of “*misleading fake news*” [61./newspaper/Feb/2020]. Questioning the mediatisation of the corona disease, Brazil’s President claimed that other serious health problems in the past “*did not cause such a commotion and repercussion from the Brazilian press*” [252./press conference/Mar/2020]. Our discursive data show that Bolsonaro repeatedly reiterated the narrative on the selective news media coverage of Covid-19 in comparison to previous infectious disease outbreaks, such as the H1N1 flu in 2009. For example, appealing to a number game rhetoric to enhance the credibility of his argumentation (Van Dijk 2006), the President insinuated that the number of cases and deaths reported by health authorities were “exaggerated” or “fake”, stating that “*last year 800 people died from that virus* [H1N1] *in Brazil and nobody said a word about it*” [482./newspaper/Mar/2020].

**Figure 1.**
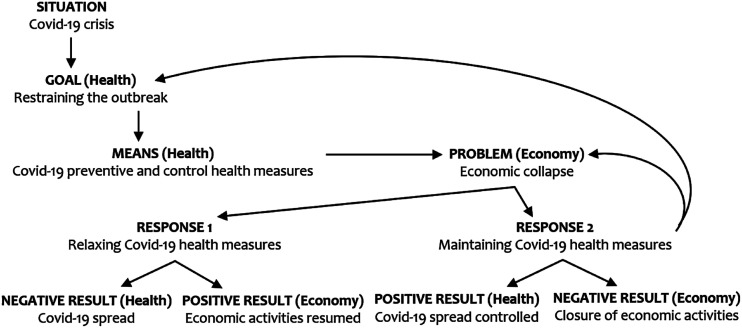
Pitting the economy against public health. Sources: Authors.

**Appealing to a false dilemma**: the antagonistic depiction of the pandemic crisis was associated with a political stalemate established between economic functioning and public health. Here the resort to such a false dilemma appeared as a means to portray Covid-19 as an economic problem caused by restrictive measures imposed by opposing political leaders, a controversial representation that helped forge divisions and heat up people’s reaction to restrictions. The source of the political impasse is that health measures needed to fight the corona outbreak were framed by Bolsonaro and his supporters as the cause of an economic crash. Such a practical argument acquires a particular significance as its underlying “representation of the causes and the nature of the crisis determines the responses to solve it” (Fonseca and Ferreira 2015:694). The circumstantial premises of Bolsonaro’s argument rested on ideas that Covid-19-related “measures will greatly jeopardise the economy”, “destroy jobs” and “lead to many more deaths”, reasons why economic crash and unemployment were depicted as “*far worse than the coronavirus itself*” [370./President/newspaper/Mar/2020]. Moreover, because a “*collapse can occur not only in health*”, imposing broad lockdown was deemed to be “*terrible for the health sector*” [334./Minister of Health 1/press conference/Mar/2020]. Thus, claims to action involved an appeal for “*getting the economy and jobs back to normal*” [511./President/TV speech/Mar/2020] by relaxing restrictive measures. As represented in Figure 1, Bolsonaro’s common-sensical appeals pitting the economy against public health (Lasco 2020) can be understood using interlocking goal-achievement and problem-solution patterns of textual organisation (Hoey 2001) present in his medical populist narrative: from a public health perspective, the pandemic situation engenders a public health goal of restraining the outbreak, then involving preventive and controlled health actions advised by national and international health authorities; from an economic view, however, the means employed to fight the pandemic replace the Covid-19 outbreak as the main reason for an economic collapse. Two opposed responses are related to the economic problem. Whereas response 1 leads to a positive economic result due to the relaxing of Covid-19-related measures, the goal of controlling the coronavirus spread is compromised. Conversely, response 2 results in achieving the public health goal of slowing the spread of the virus while leading to a negative result for the economy due to continuing social distancing. Therefore, in such a simplistic causality reasoning positive result for public health is overridden by an immediate negative result for the economy, prompting a recycling of the economic problem.

### Overpoliticisation of all dimensions of the health problem

**Undermining conventional approaches**: the antagonistic depiction of the pandemic constructed through minimisation, redramatisation and impasse-driven narratives, contributed to the strong politicisation of major health strategies implemented. Generally speaking, politicising is a process of making all questions political questions, all problems political problems. This entails exposing and questioning what is assumed “as morally or politically obligatory and essential” (Jenkins 2011:159), which may help strengthen the political responsiveness of public administrations (Hustedt and Salomonsen 2014). Although politicisation is not inherently negative (Hart et al. 2020), overpoliticisation in health crises may indicate a crisis of expertise (Bal et al., 2020) and a decline of the authority of science (Gauchat 2012) in providing sufficient legitimacy to professional bureaucracies and expert policymakers. In Brazil’s Covid-19 crisis, high politicisation expressed the expanded influence of populist leaders who, in the process of competing for power (Krzyżanowski, Triandafyllidou and Wodak 2018), articulated medical populist discourses to dispute more traditional, technocratic responses (Lasco 2020). In that expert knowledge became overwhelmed by political orientations, such overpoliticisation of the coronavirus emergency created an imbalanced arena of power and agency by shifting many technical aspects of the health problem to the political domain. This not only greatly increased the pressure of populist discourses and interests over the authority of bureaucrats and scientific advisors but also exacerbated partisan divisions and views on the issue:

667. […] Brazil had time to prepare, but **there was politicisation of the pandemic and that jeopardised the country greatly.** […] we didn’t manage because **politicisation undermined the fight against Covid-19** […]. [Director of the Department of Immunization and Transmissible Diseases of the MH/newspaper/Apr/2020]

687. He [the President] clearly expresses that **he wants another stance for the MH.** I have this path to offer, **based on science.** Out of this path, other alternatives have to be found. [Minister of Health 1/press conference/Apr/2020]

813. We cannot turn this [lockdown] into a **political discussion.** This is a technical discussion. [Minister of Health 2/newspaper/May/2020]

**Figure 2.**
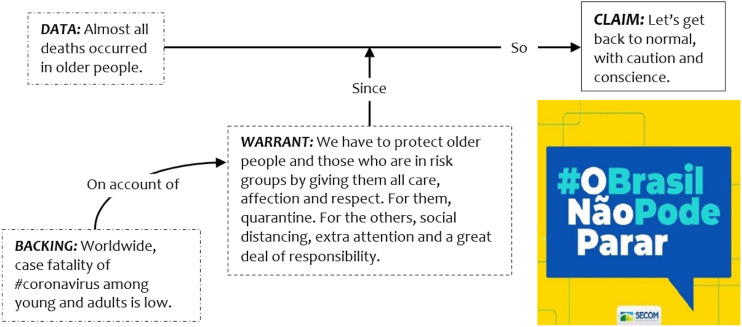
The #BrazilCannotStop advertising campaign. Source: Authors.

**Underrating drastic measures**: closely associated with the theme above, the medical populist discourse built on the rhetoric of downplaying the severity of the epidemic to construct a narrative focused on underrating drastic contingency interventions advocated by technical advisers, academics and most local governments, while fostering the need to keep economic activities open, as observed in the federal government’s ad campaign “#BrazilCannotStop” (see Figure 2). Launched by the President’s communication department, such a campaign is an example of hortatory discourse, a common genre in government policy formation (Fairclough 2003), with an explicit prescriptive intent aimed at persuading the public of certain policies. As Toulmin’s (2003) argumentation model clarifies, Bolsonaro’s campaign to “get back to normal” relied on the warrant that more drastic measures (e.g. lockdown, quarantine) were only needed for at risk groups, understood to be older adults and people with health conditions, and therefore less restrictive measures could be applied to the rest of the population. This warrant was in turn grounded in preliminary evidence that most deaths occurred in older people.

**Figure 3.**
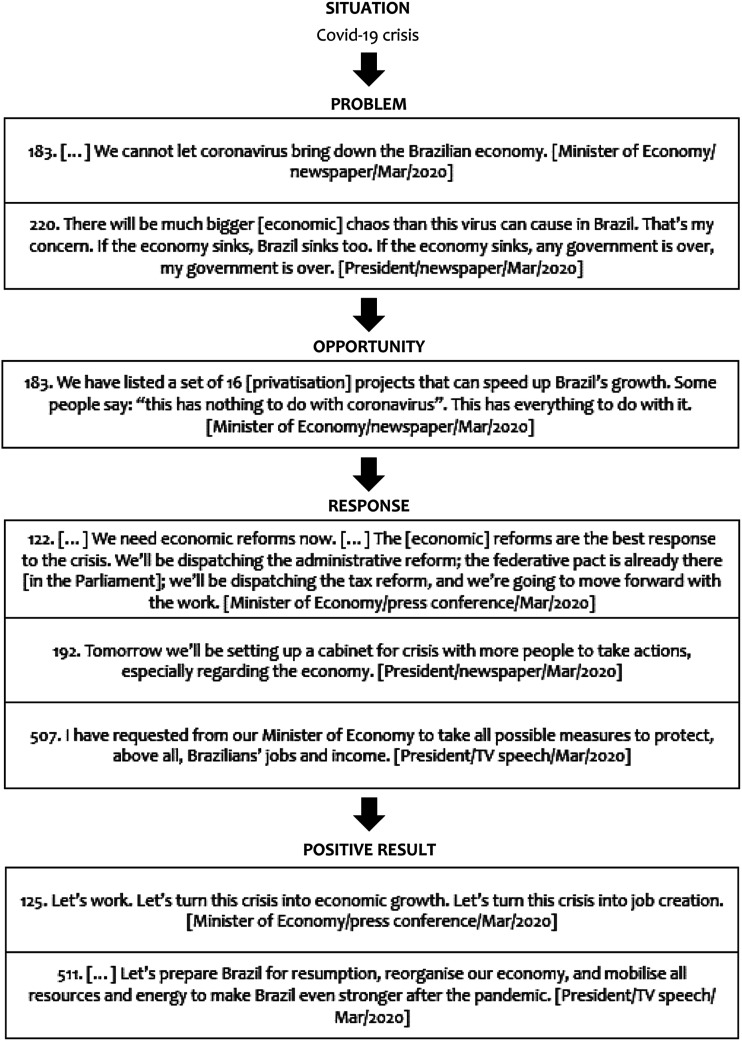
Discursive patterns of prioritisation of economy (examples). Sources: Authors.

**Prioritising the economy**: differing from a strategy designed to balance epidemic control and economic functioning (Cheng et al., 2020), Bolsonaro’s government prioritised the economy over more drastic health policies. The prioritisation of economic objectives is expressed in a practical argumentation that combines a problem-solution and opportunity-taking textual frames (Hoey 2001) in which Covid-19 is conceived in terms of an economic problem and the response as deriving from opportunistically advancing the economic reform agenda, including privatisation, administrative and tax reforms, to enable the government to protect “Brazilians’ jobs and income”. This opportunity-taking frame is completed with expected positive results that signal a turning point in “economic growth”, “job creation”, and “resumption” to make “the country even stronger” (see Figure 3). Discursive legitimation strategies helped justify the prioritisation of efforts to resume economic activities. Legitimation plays a key role in the structure of argumentation of a political speech, because, through legitimating discourses, political speakers seek to provide good reasons, reasonable and acceptable motivations (Van Dijk 1998; Fairclough and Fairclough 2012) that not only legitimise intended policies and decisions but also help delegitimise alternative options (Fonseca and Ferreira 2015). Our data show that medical populist discourses not only spoke out against but can also drew on technocratic institutions to gain legitimation.

A major strategy of legitimation employed by President Bolsonaro involved a call for an institutional authority (Fairclough 2003; Van Leeuwen and Wodak 1999) to reinforce the idea that people needed to get back to work for their daily bread. By analysing direct and indirect reporting as key aspects of intertextuality, or “how texts draw upon, incorporate, recontextualise and dialogue with other texts” (Fairclough 2003:17), we found that Brazil’s President directly resorted to selected quotations from the WHO Director-General, Tedros Adhanom, stressing the economic implications of measures that restrict the movement of people, to establish a positive dialogical relation between Bolsonaro administration’s claims for getting back to work and the concerns mentioned by the head of the WHO. Less emphasis, however, was given to passages about governmental social policies as advised by Adhanom to support people and enable them to comply with Covid-19 health measures:

505. Protective measures must be implemented in a rational, responsible and coordinated way. In this sense, Mr Tedros Adhanom, WHO Director-General, has said he acknowledges that **“many people, indeed, have to work every single day to win their daily bread”** and that “governments have to take these people into account”. He continues: **“If we're closing or if we're limiting movements what is going to happen to those people who have to work on a daily basis and have to earn their bread on a daily basis?”.** [President/TV speech/Mar/2020]

513. Have you guys seen what the WHO Director-General has said? Has anyone seen that? He basically has said, especially concerning informal workers: **“they gotta work”.** [President/newspaper/Mar/2020]

516. […] We understand that many countries are implementing measures that restrict the movement of people […]. **Governments need to ensure the welfare of people who have lost their income and are in desperate need of food, sanitation and other essential services.** [WHO Director-General/press conference/Mar/2020]

521. People without regular incomes or any financial cushion deserve **social policies that ensure dignity and enable them to comply with #COVID19 public health measures** advised by national health authorities and @WHO.[WHO Director-general/Twitter/Mar/2020]

**Claiming simplistic solutions**: our data suggest that it is crucial for medical populism to be able to offer alternative solutions in order to sustain its opposing stance towards more orthodox approaches of policymaking and implementation. In this case, proposing controversial quick fixes is part of the overpoliticisation narrative, usually backed by common sense, first-hand experience or limited scientific evidence (Lasco and Curato 2019; Brubaker 2017). Table 2 explores the notion of relational processes to show how contradictory views around simplistic solutions like “vertical isolation approach” to contain the outbreak and the “use of (hydroxy)chloroquine” to treat infected patients were discursively framed in political actors’ narratives. As Halliday and Matthiessen (2004) explain, discursive relational processes refer to how processes of being and having are characterised and identified through language grammar. Discourses on vertical isolation and (hydroxy)chloroquine were analysed according to two modes of relational processes: (i) attributive, in which one entity (ATTRIBUTE) is ascribed to another (CARRIER), and (ii) identifying, in which one entity (VALUE) is being used to identify another (TOKEN). For example, quite antithetic attributes were employed in the framing of the extended use of (hydroxy)chloroquine to treat Covid-19 patients, such as “effective” by the President and “panacea” by his first Minister of Health. By identifying this medicine as “the cure” for the corona disease, Bolsonaro’s cabinet office instructed the army to expand the national production of chloroquine for treating and preventing Covid-19, even in the absence of scientific advice from the MH and the National Health Surveillance Agency (ANVISA). To legitimise this decision, the federal government cited a technical report from the Brazilian Federal Council of Medicine (CFM) about the broader use of (hydroxy)chloroquine in Covid-19 cases. The CFM here suggested considering “*the use of chloroquine and hydroxychloroquine to treat Covid-19 under exceptional circumstances*” [724./CFM Report n. 4/2020/Apr/2020], on the basis of limited studies about the efficacy of these medicines in treating Covid-19 infection. The Minister of Health 2, Nelson Teich, decided to step down after warning against (hydroxy)chloroquine side effects and refusing the government cabinet request to put more flexible regulating protocols in place to allow the use of these medicines in patients with milder forms of SARS-CoV-2. Likewise, the discursive examples illustrate that while some actors framed vertical isolation as “the ideal” approach for quarantine, others pointed out its “weaknesses” in being considered a “definitive solution”. Proposed by the cabinet office, this vertical isolation framework recommended quarantine only for risk groups, but did not find support from the MH. Eventually, the government pressure for vertical isolation, the request to ease (hydroxy)chloroquine regulating protocols, and differing opinions about essential services to remain open aggravated internal disagreements between the government cabinet and the MH, leading Bolsonaro to appoint a close allied general, Eduardo Pazuello, to take up the post as Brazil’s health minister.

**Table 2.** Contradictory discourses on vertical isolation and (hydroxy)chloroquine.

Attributive relational process CARRIER + process + ATTRIBUTE	Identifying relational process TOKEN + process + VALUE
574. […] such a strategy [vertical isolation]**has** also weaknesses and **does not constitute** a definitive solution to the problem. [Minister of health 2/LinkedIn article/Apr/2020] 509. […] although hydroxychloroquine**seems** quite effective. [President/TV speech/Mar/2020] 594. The use of chloroquine**has** increasingly **been presented** as something effective […]. [President/Twitter/Apr/2020] 470. That medication, chloroquine, **is working**well everywhere. [President/newspaper/Mar/2020] 464. Chloroquine**is not** a panacea […]. [Minister of health 1/press conference/Mar/2020] 371. That medicine [chloroquine]**has**strong side effects […]. [Minister of health 1/newspaper/Mar/2020] 865. An important warning: Chloroquine**has**side effects […]. [Minister of health 2/Twitter/May/2020] 479. […] studies [on the efficacy of (hydroxy)chloroquine for Covid-19] **are** still insufficient. [Secretary of science, Technology and strategic Supplies of the MH/newspaper/Mar/2020]	403. […] from now on the advice**is going to be**vertical [isolation]. [President/newspaper/Mar/2020] 681. […] the vertical isolation**is**the ideal now. [Minister of Women, family, and Human Rights/YouTube channel interview/Apr/2020] 573. […] the choice for horizontal isolation, […], **is**the best strategy at this moment. [Minister of health 2/LinkedIn article/Apr/2020] 469. […] [hydroxychloroquine] **is being used**in several hospitals. […] the cure**is**there. [President/newspaper/Mar/2020] 464. […] [the chloroquine]**is not**the medicine that came to save mankind. [Minister of health 1/press conference/Mar/2020]

Source: Authors.

### Moral interpretation of political actors

**Drawing on normative polarisation**: as suggested by Osuna (2021), populist movements often entail a moral or normative interpretation of political actors as opposed to an empirical one, with an emphasis on an antagonistic depiction of the “pure, virtuous people” and the “corrupt elite” (Mudde 2010; Laclau 2005). Our findings indicate that establishing a normative polarisation among central and local political leaders was a key feature of the medical populist discourse on the Covid-19 crisis, contributing to creating an equivalence chain to canalise unsatisfied popular demands and mobilise part of the population, or “the people”, frustrated with economically detrimental restrictive measures. One normative polarisation structure underlying such medical populist discourse refers to the US vs THEM dichotomisation frame based on how political actors and policymakers were interpreted either as leaders who interpellated the underdog or a perceived common enemy or threat. As illustrated in Table 3, for instance, debates on Covid-19 policy in Brazil signal medical populist narratives intensely demarcated by symbolical frames of positive self-presentation and negative other-presentation (Van Dijk 2006) in which political speakers emphasise the positive actions of their own group (e.g. providing working conditions, avoiding panicking) while accentuating the negative actions of rival groups (e.g. jeopardising the economy, downplaying the pandemic). The US vs THEM dichotomisation also involved the application of narratives of blame avoidance by the ingroup and blame allocation to the outgroup. As Hinterleitner (2017) explains, political actors seek to avoid responsibility for bad outcomes of government decisions either by keeping themselves away from potentially blame-generating and goal-threatening events or by shifting blame to other actors or entities. For example, President’s narratives to avoid blame-attracting pressures involved the argument that he could not interfere in certain things given the Supreme Court’s decision about local governments’ prerogatives to act upon the corona crisis in conjunction with the federal government. At the same time, the President repeatedly identified local authorities, international health bodies and parts of the media as blame-eligible individuals who should be deemed morally accountable for economic crisis and unemployment. Bolsonaro and his followers also tried to construct a positive moral interpretation of their actions and views by exploiting values, emotions and beliefs which they associated with projected positive outcomes of the proposed easing of restrictions (see Table 4). This normative framework served to legitimize government actions through moral evaluations (Fairclough and Fairclough, 2012) that helped increase the acceptability (Fonseca and Pereira, 2015) of its political arguments and decisions. In this light, we argue that polarisation reflected the operation of a logic of equivalence (Laclau and Mouffe 1985) in medical populism on the Covid-19 crisis in Brazil, “a dichotomization of the political spectrum through the emergence of an equivalential chain of unsatisfied demands” (Laclau 2005:74) the populist leaders exploited to construct a categorical division of subjects and a seemingly unbridgeable gap between the ingroup (US) and the outgroup (THEM) (Van Dijk 2006).

**Table 3.** The US x THEM polarisation.

	***Emphasising OUR good actions***	***Emphasising THEIR bad actions***	
**POSITIVE SELF-PRESENTATION**	90. […] the president **has provided us with all conditions** to work. [Minister of health 1/newspaper/Mar/2020] 191. I would like them [other political leaders] to go out onto the streets **as I did**. [President/TV interview/Mar/2020] 368. **We are in direct contact with mayors […]. That’s our job: **to calm people down and avoid panicking, […]. [President/newspaper/Mar/2020] 445. **We didn’t take any measure without discussing** it with the scientific community, researchers, doctors and health professionals. [Governor of goiás/newspaper/Mar/2020] 876. Since the beginning of this pandemic in Brazil, president bolsonaro **has always been concerned with balancing actions** in health and economy. [Minister of citizenship/newspaper/May/2020] 877. The president **has provided the health area with all conditions** to strengthen the SUS […]. **Support has been given** to governors and mayors. [Minister of citizenship/newspaper/May/2020]	241. […] there are some governors who are, in my view, I might be wrong, **taking measures that will greatly jeopardise our economy**. [President/newspaper/Mar/2020] 410. **It’s a crime what a few governors and mayors have been doing to Brazil. They are** wrecking the country and destroying jobs. [President/newspaper/Mar/2020] 824. I’ve been seeing many mayors complaining because **they want their cities back to work but the respective governor does not allow it**. [President/newspaper/May/2020] 228. Over here, the president of the republic **ignores and undermines** his minister of health and experts of the ministry by **downplaying the pandemic and encouraging people to go out on to the streets** […]. [President of the house of Representatives/Twitter/Mar/2020] 235. […]He [the president] should be the first one to set a good example, **not a bad example as he did**. [Governor of São paulo/press conference/Mar/2020] 653. It’s not appropriate that the president adopts a behaviour that **contradicts his own government** [advice]. [Mayor of Manaus-AM/newspaper/Apr/2020]	**NEGATIVE OTHER-PRESENTATION**
	***De-emphasising OUR bad actions*** 919. Today Brazil celebrates 100K saved lives amid the world crisis. […] **while many put emphasis on death, the brazilian government works for life and celebrates life**. [Special Secretariat for social Communication/Twitter/Jun/2020] 838. […] the virus hasn’t changed and won’t change our values. We’re going to stand our ground. **The enemy of the economy is not the quarantine, it’s the virus** […]. [Governor of São Paulo/Twitter/May/2020]	***De-emphasising THEIR good actions*** 188. […] certainly, some people want to **create a smokescreen to cover their work** which is not being approved by part of the population. [President/TV interview/Mar/2020] 927. Transparency’s VICTORY: the STF [Supreme court] has accepted our request and broken Bolsonaro’s sanitarian pedalling. **The government will be forced to publicise data about the pandemic** as before, without making-up or manipulation. [Senator/Twitter/Jun/2020]	
**BLAME AVOIDANCE**	***Keeping US away from blame-generating pressures*** 223. There are certain things you cannot interfere in. **I can’t interferein it** […]. [President/radio interview/Mar/2020] 363. Do not exterminate jobs, Messrs., governors. Be responsible. **I hope they don’t try to blame me for millions and millions of unemployed people in the future**. [President/TV interview/Mar/2020] 825. **I’m not fiddling with anything**. Health is life. The decision to close businesses is up to the respective governor or mayor. **I’ve been doing what is my responsibility**. [President/newspaper/May/2020]	***Shifting blame over to THEM*** 345. I Am concerned about people’s lives and **unemployment caused by these negligent governors**. [President/newspaper/Mar/2020] 367. The people will soon see that **they were tricked by these governors and by the large part of the media** when it comes to coronavirus. [President/newspaper/Mar/2020] 613. […] if you have any issue with the state or think that quarantine and measures taken by your state are prejudicing you, **the right jurisdiction to complain to is your respective governor or mayor**. [President/YouTube channel/Apr/2020]	**BLAME ALLOCATION**

Source: Authors.

**Table 4.** Legitimation by reference to a normative framework.

Values, emotions and beliefs exploited		Projected positive outcomes
Challenge Concern Fear Distress hard work Rationality Responsibility Coordination Commitment Collaboration	Courage Support Safeguard Cautiousness Precaution Empathy Caring Saving lives Solidarity Unity	Preserving families’ subsistence Saving lives taking care of people’s health Saving jobs fighting unemployment Protecting income job creation Economic growth

Source: Authors.

**Adopting a confrontational approach**: nourished by a self-image of exclusive defenders of the common good (Müller 2017) and guided by a moralistic rather than programmatic agenda (Osuna 2021), Bolsonaro’s administration proposed a non-linear approach to avoid broad lockdown, an idea that came into conflict with rival political authorities who had ordered more restrictive safety measures to fight local spread. On the one hand, governors and mayors were criticised by the President for imposing unacceptable restrictive measures, lexicalised as “*scorched-earth*” policies [383./President/TV speech/Mar/2020] in his moral panic repertoire (Lasco and Curato 2019). On the other hand, the deficient articulation of governmental efforts was expressed in local authorities’ refusal to embrace Bolsonaro’s approach of rolling back arrangements that were limiting social functioning. As stressed by the Governor of Rio de Janeiro, for example, “*there’s no sign of easing restrictive measures. It is irresponsible to encourage entrepreneurs to reopen their businesses*” [841./Twitter/ May/2020]. Similarly, the President of the Senate declared that “*it is reckless to encourage people gatherings on the streets*” [233./Twitter/Mar/2020]. Controversies became even more critical given that Bolsonaro rules within a politico-institutional system that limits his policy authority (Barberia and Gómez 2020). According to the Brazilian Constitution, municipalities, states and the federal government have concurrent responsibility for public health. Nevertheless, contrasting policy orientation from Bolsonaro’s government led the Federal Council of the Brazilian Bar Association (CFOAB) to file a suit in the Brazilian Supreme Court to question “*omissive and commissive acts performed by the federal Executive Power in the context of the public health crisis caused by the Covid-19 (coronavirus) pandemic*”. Aiming at upholding governors’ and mayors’ health policies and solving “*[…] the serious divergence of stances among authorities of different federative levels*” [605./Lawsuit against Noncompliance with Fundamental Precept/ADPF n. 672/2020/Apr/2020], the Supreme Court decided that:

611. […] **the federal Executive Power cannot unilaterally rule out decisions of state, district and municipal governors that**, in the exercise of their constitutional functions, **have adopted or may come to adopt significant restrictive measures** […] **recommended by the WHO (World Health Organization) and several technical and scientific studies** […]. [Lawsuit against Noncompliance with Fundamental Precept/ADPF n. 672/2020/Apr/2020]

As Lasco (2020) notes, the politics of pandemics involve a complex interplay of actors, which is why the success of a policy to fight an outbreak cannot be ascribed to central political leaders only. Public and community-based organisations are also crucial for successful policies (Cheng et al., 2020). Likewise, trustworthy and efficient communication are of the utmost importance for effective crisis management (Christensen and Lægreid 2020). Nevertheless, our data illustrate that in the Brazilian corona crisis poor collaborative governance between Bolsonaro administration and regional authorities and a lack of coordination within the federal government itself were decisive discursive frames in eroding citizens’ trust and compliance (see Table 1).

## Discussion and conclusion

Employing CDA, we found that the main discursive frames underpinning medical populism during the Covid-19 crisis in Brazil reflected the most widely agreed attributes of populism as a strategic political discourse (Osuna 2021; Van Dijk 2006), notably an antagonistic depiction of the health problem, high politicisation and moral interpretation of political actors. Our study corroborates extant research suggesting that health crises can be used as a canvas for populist performances (Lasco and Curato 2019), making medical populism a performative form of political discourse that populist leaders draw on to reach or exercise power in the framing of public health policy and management. Our findings also challenge some theoretical assumptions of extant conceptualisations of medical populism, thus contributing to the identification of distinct discursive features and narratives within this phenomenon.

Medical populism rests on the antithetical representation of “the people” versus “the establishment”, “the dominant power structure” or, simply, “the elite” (Lasco and Curato 2019; Canovan 1999; Laclau 2005). Indeed, medical populist discourses on the corona crisis in Brazil worked by deepening existing symbolical and political divisions in the national politics, government and policymaking, exacerbating an already highly polarised political environment and jeopardising inter-governmental action for health. As a discursive ramification of President Bolsonaro’s anti-establishment rhetoric, medical populism manifested as a sort of reaction, negativism or counter-hegemonic endeavour to cultivate the aspirations of “the underdog” and challenge “the system” (Osuna 2021) whose failures or hidden interests (Lasco and Curato 2019) have allegedly led to health and economic crises. In line with previous research, the findings presented here suggest that medical populism serves as a discursive strategy for wider political ends pursued by populist leaders (Speed and Mannion 2020). Seen as an archetypal voice of contemporary rightist post-truth politics, Bolsonaro sought to build on medical populism to mobilise people’s prejudices, fears, anxieties and frustrations (Jenkins 2009; Müller 2017) with Covid-19 restrictive interventions and to elicit a public reaction (Ungar 2001) against the political and socio-economic elites (Osuna 2021).

However, our findings suggest that the discursive frames inscribed in medical populist claims to construct an antagonistic depiction of the crisis may express meanings and schemes of argumentation that contrast with extant literature. For example, whilst previous research into medical populism sustains that moral panics (Cohen 2011) integrate the discursive repertoire of populist leaders who seek to promote hysteria “by exaggerating threats to public health” (Lasco and Curato 2019:3) in order to ask for drastic changes (Osuna 2021), in the context of our study medical populism was articulated under a narrative of minimising the pandemic crisis. The scepticism underlying such a discursive frame was based on the conspiratorial idea that institutions, public authorities and professional experts claiming more draconian measures to fight coronavirus have become instruments of a corrupt system and globalist order that obstruct people’s will and freedom. So, by minimising the pandemic through the use of pejorative lexicalisation and naturalisation of certain presuppositions regarding the corona disease, Bolsonaro and his supporters were able to carve out a discursive frontier that reinforced their sense of moral superiority and set them apart from the representatives of “the system” (Osuna 2021; Müller 2017).

Medical populism presupposes an appeal to “the people”, pitted against “the dominant order of power” on account of threats to the public’s health and safety rather than cultural and economic insecurity, which are most common in other forms of populism (Lasco and Curato 2019; Speed and Mannion 2020). In our case study, however, President Bolsonaro’s discourses built on a dilemma between health threats and economic turbulence both to minimise the severity of the health crisis – while defending scientifically unsubstantiated solutions like vertical isolation and (hydroxy)chloroquine and adhering to anti-vaccine movements – and to advance economic reforms. Medical populism, in this case, expressed the precedence of the economy over public health in the framing of policy actions, hence greatly influencing local governments to move on to Stage Four of the national health response (i.e. relaxing restrictions and resuming social functioning). In this light, the Brazilian case indicates that in times of economic stagnation and rising public debt medical populism can be used to subordinate the health crisis to economic projects (e.g. neoliberal reforms) while feeding other common right-wing populist narratives against perceived economic threats (e.g. communism, big government and waste, globalism, etc.). This highlights Mudde’s (2010) idea that populism is never pure but rather blends with other ideologies, discourses and political strategies.

The Brazilian case is also intriguing because medical populism embedded in Bolsonaro’s discourses drew upon the crisis not to legitimise immediate and energetic actions, as Moffitt and Tormey (2014) would predict, but to perpetuate the pandemic emergency while adopting demagogic approaches such as “enabling the people to earn their daily bread”. This served as a pretext for emphasising the functioning of the economy, seen as most vital to maintain the government’s popularity. Whereas populist leaders often dramatise, amplify and distort crisis episodes (Brubaker 2017) through appeals to moral panics (Cohen 2011) in order to obtain emergency powers for imposing exceptional health policies (Lasco 2020), redramatising the pandemic in Brazil was a sort of “reverse panicking narrative” used in medical populist discourses to claim panicking was being caused by the media and opposing political actors. Interestingly, Bolsonaro’s criticism of mediatisation and spectacularisation of the corona crisis in Brazil did not reflect an attempt to “dedramatise” the health crisis aiming to assuage public fears but to “redramatise” it as a means to mobilise followers and gain political support against restrictive interventions implemented by local governors.

Our discourse analysis endorses the assumption that overpoliticisation of public administration is a central feature of medical populism (Lasco and Curato 2019), highlighting how medical populist discourses on Covid-19 went hand in hand with a sort of discretion-based governance (Wood 2016) of the epidemic. More than simply denoting a mechanism adopted by the government to facilitate putting political decisions into action (Peters and Pierre 2004), this type of governance reflected the denial of the authority of science (Gauchat 2012) and the defence of simplistic solutions (Moffitt and Tormey 2014) against the advice of established institutions and professional bureaucracies. Such mode of statecraft involved discursive manoeuvres aimed at expanded preference shaping strategies to prioritise economic goals to the detriment of more conventional approaches to medical emergencies. This movement was particularly strong during the early stages of the epidemic in the country when Bolsonaro’s narratives placed greater emphasis on propagating a general distrust of the complex apparatus of administration and policymaking in order to supplant drastic evidence-based measures with unfounded short-term and rapid responses (e.g. (hydroxy)chloroquine, vertical isolation).

The intensity of politicisation has been remarkable throughout the pandemic in Brazil. However, such a governance strategy tends to recede and take other forms in the face of public criticism and resistance from the medical professional establishment. Our data reveal that public perceptions of poor governance and inter-governmental collaboration put pressure on Bolsonaro’s administration to seek support from expert advisers. Eventually, this led Bolsonaro to reshape and utilise health bodies of the federal government as instruments to achieve his ideals by exploiting a strategy of “professional politicisation” (Perters 2013:17), that is, the appointment of politically sympathetic yet highly professionalised experts from the upper echelons of the civil service to key positions in public health institutions (e.g. ministers of health, agency executives). This highlights how the complex, uneasy and multifaceted interface between politics and public management may evolve in a health crisis context pervaded by populism, exemplified by the constant reshuffle of government ministers and public officials, sometimes to present a picture of reduced political influence on public decision-making, sometimes to amplify direct ministerial coordination in order to fuel antagonisms and foster soundbite solutions.

As Gostin, Moon and Meier (2020:1615) note, the Covid-19 pandemic “is reframing global health governance”. This is particularly so in the context of rising radical populist nationalism. Although this study is nation-specific, the Brazilian case indicates that medical populism may affect public trust, solidarity and disposition to adopt internationalist foreign policy orientations (Bayram and Shields 2021) for managing common health threats. For example, our analysis illustrated some of the political vulnerabilities that national and international health agencies may be subject to within pandemic politics polarised by medical populist lines (Lasco and Larson 2020). Those agencies – the WHO, in particular – tended to be discursively singled out as blame-eligible institutions associated with globalist interests rather than prestigious and trustworthy organisations able to coordinate cross-national policies to address the crisis.

Nonetheless, our study suggests that geopolitical health organisations may not only be attacked by populist discourses but also cunningly exploited to legitimise certain policy actions through appeals to institutional authority. According to our data, even though overpoliticisation promoted by populist rhetorical practices is commonly accompanied by an explicit anti-intellectualism, negativism and anti-globalisation (Lasco 2020; Osuna 2021), it can also be softened to attract support from professional experts linked to stable national and international policy communities (Peters 2013). This was crucial for providing a certain degree of “epistemological” legitimation to medical populist discourses and solutions proposed by Bolsonaro’s government. In some sense, therefore, high politicisation of epidemics may make room for bringing senior political leaders closer to expert opinion leaders who are able to offer political-tactical advice (Hustedt and Salomonsen 2014) on policy agenda setting and implementation.

Medical populism tends to perform around logics of equivalence to demarcate ingroups from outgroups (Speed and Mannion 2020). It is, therefore, a performative political discourse that becomes evident wherever the logic of equivalence triumphs over the logic of difference (Moffitt and Tormey 2014). We further extend this argument by showing how medical populism employed a normative framework to lend an aesthetic and emotional nature (Lasco and Curato 2019) to the Covid-19 health emergency in Brazil, thus facilitating the construction of political identity through (Laclau 2005) moral interpretation of political actors involved. This in turn helped Bolsonaro and his supporters to carve out a normative polarisation through which they sought to (re)shape policy proposals, unite and evoke reactions from a targeted audience of followers and deflect blame for policy failure (Wood 2016). The Brazilian example illustrates how medical populism followed a logic of equivalence to forge a US vs THEM dichotomisation through discursive frames of positive self-presentation and negative other-presentation. These frames were a mechanism to idealise and aestheticise confrontation (Osuna 2021) by establishing a protagonist‒antagonist relation between political actors linked to Bolsonaro’s politics, on the one hand, and opposing political leaders, technocrats and academics, on the other. By promoting such a logic of equivalence, medical populist discourses made the Covid-19 response a source of social conflict and ideological and party-political disputes, where medical populism competed with, and indeed undermined, conventional policy approaches that recommended more restrictive health measures.

In this vein, our discursive analysis contributes to the literature on the performativity of medical populist narratives based on a confrontational approach, inflammatory rhetoric and popular resentment against “established power structures” (Canovan 1999; Speed and Mannion 2017; Lasco and Curato 2019; Lasco 2020) by demonstrating the ways other responses (e.g. rationalist, technocratic, EBM, etc.) may be argumentatively subverted. Although Brazil’s public healthcare system counts on highly institutionalised and trusted professional bodies that tried to develop a more rationalist, evidence-based health policy, the corona crisis clearly illustrates how medical populist discourses may be effective in employing post-truth politics through social media to frame health issues, subvert expert opinions and legitimise alternative solutions, thus leading to misguided decisions and public’s low compliance with preventive measures defined by national and international health agencies.

In conclusion, this study provides greater insights into the concept of medical populism by exploring how this type of political discourse may incorporate different discursive meanings, structures and schemes of argumentation into its populist repertoire. This work also adds to the growing discussion about Covid-19 in the field of public policy and administration. The Brazilian experience offers lessons to national and international policymakers, practitioners, academics and general readers facing the current pandemic, especially those engaged in health policy issues and debates around the economic and social implications of restrictive public policies targeted at coronavirus mitigation.

Firstly, political discourses on the corona crisis in Brazil endorse the idea of the global extent of medical populist performances (Lasco 2020) not as an “episodic but a familiar response to medical emergencies” (Lasco and Curato 2019:6) which plays a decisive role in framing public policy development and implementation. Considering that this trend has important implications for the processes and political dynamics surrounding communication and governance of pandemics in national and global contexts, our CDA approach provides quite illustrative examples of the sorts of challenges that may be faced by policymakers around government communication, widespread misinformation, and declining credibility of science, medicine and public health institutions. In this vein, the Brazilian case highlights the potential of medical populism to increase the public’s propensity to favour political over scientific viewpoints, leading them to disregard information that does not support their views, even when information is provided by professionals or expert health bodies (Hart et al. 2020).

Finally, our case study shows that even healthcare systems that are grounded on well-developed networks of participatory governance and decentralised decision-making, such as the Brazilian SUS, can be destabilised by medical populism, especially at the early stages of a health crisis. This kind of populist performance may induce poor collaborative working and multilevel governance (Ongario, Gong and Jing 2018) among different spheres of government and policy actors responsible for (re)framing and translating policy into practice, thus raising questions of authority, risk governance, blame-shifting and self-preservation strategies (Lasco 2020; Dunlop et al. 2020).

Therefore, the analysis of medical populist discourses may help political and professional actors engaged in pandemic politics understand the rationales behind the populist turn in public policy and administration as well as its implications for health policy change, decision-making and management. Such an understanding seems imperative to anticipate patterns of action and narratives for preparing responses to future public health emergencies at local, national and international levels (Lasco 2020), as populist discourses seem likely to influence public policy and governance for some time.

## Supplemental Material

Supplemental Material - ‘This is just a little flu’: analysing medical populist discourses on the Covid-19 pandemic in Brazil Click here for additional data file. Supplemental Material for ‘This is just a little flu’: analysing medical populist discourses on the Covid-19 pandemic in Brazil by Erik Persson, Ewan Ferlie, and Juan Baeza in Public Policy and Administration

## References

[bibr1-09520767221141121] Almeida F (2021) Exploring the Impact of COVID-19 on the Sustainability of Health Critical Care Systems in South America. International Journal of Health Policy and Management 10(8): 462–464. 32654433 10.34172/ijhpm.2020.116 PMC9056199

[bibr2-09520767221141121] Bacchi C (2000) Policy as Discourse: What Does It Mean? Where Does It Get Us? Discourse: Studies in the Cultural Politics of Education 21(1): 45–57.

[bibr3-09520767221141121] Bal R Graaf B Bovencamp HV, et al. (2020) Practicing Corona–Towards a research agenda of health policies. Health Policy 124: 671–673. 32425282 10.1016/j.healthpol.2020.05.010 PMC7228690

[bibr4-09520767221141121] Bayram AB Shields T (2021) Who Trusts the WHO? heuristics and Americans’ trust in the world health organization during the COVID-19 Pandemic. Social Science Quarterly 102(5): 2312–2330. 34226772 10.1111/ssqu.12977 PMC8242889

[bibr5-09520767221141121] Barberia LG Gómez EJ (2020) Political and institutional perils of Brazil’s COVID-19 crisis. The Lancet 369(10248): 367–368. 10.1016/S0140-6736(20)31681-0 PMC7392557 32738938

[bibr6-09520767221141121] Brubaker R (2017) Why populism? Theory and Society 46: 357–385.

[bibr7-09520767221141121] Canovan M (1999) Trust the People! Populism and the Two Faces of Democracy. Political Studies 47(1): 2–6.

[bibr8-09520767221141121] Cheng Y, et al. (2020) Coproducing Responses to COVID-19 with Community-Based Organizations: Lessons from Zhejiang Province, China. Public Administration Review 80(5): 866–873. 32836448 10.1111/puar.13244 PMC7283761

[bibr9-09520767221141121] Christensen T Lægreid P (2020) Balancing governance capacity and legitimacy–how the Norwegian government handled the COVID-19 crisis as a high performer. Public Administration Review 80(5): 774–779. 32836445 10.1111/puar.13241 PMC7280699

[bibr10-09520767221141121] Cohen S (2011) Folk Devils and Moral Panics. London: Routledge.

[bibr11-09520767221141121] Dunlop CA Ongaro E Baker K (2020) Researching COVID-19: A research agenda for public policy and administration scholars. Public Policy and Administration 35(4): 365–383.

[bibr12-09520767221141121] Fairclough N (2003) Analysing Discourse: Textual Analysis for Social Research. London: Routledge.

[bibr13-09520767221141121] Fairclough N Fairclough I (2012) Political Discourse Analysis: A Method for Advanced Students. London: Routledge.

[bibr14-09520767221141121] Fischer F (2003) Reframing Public Policy: Discursive Politics and Deliberative Practices. Oxford: Oxford University Press.

[bibr15-09520767221141121] Fonseca P Ferreira MJ (2015) Through ‘seas never before sailed’: Portuguese government discursive legitimation strategies in a context of financial crisis. Discourse & Society 26(6): 682–711.

[bibr16-09520767221141121] Garvin T Eyles J (2001) Public health responses for skin cancer prevention: the policy framing of Sun Safety in Australia, Canada and England. Social Science & Medicine 53(9): 1175–1189. 11556608 10.1016/s0277-9536(00)00418-4

[bibr17-09520767221141121] Gauchat G (2012) Politicization of Science in the Public Sphere: A Study of Public Trust in the United States, 1974 to 2010. American Sociological Review 77(2): 167–187.

[bibr18-09520767221141121] Gostin LO Moon S Meier BM (2020) Reimagining global health governance in the age of COVID-19. American Journal of Public Health 110(11): 1615–1619. 33026872 10.2105/AJPH.2020.305933 PMC7542258

[bibr19-09520767221141121] Halliday M Matthiessen C (2004) An Introduction to Functional Grammar. New York: Oxford University Press.

[bibr20-09520767221141121] Hart PS Chinn S Soroka S (2020) Politicization and Polarization in COVID-19 News Coverage. Science Communication 42(5): 679–697. 10.1177/1075547020950735 PMC7447862 38602988

[bibr21-09520767221141121] Hinterleitner M (2017) Reconciling perspectives on blame avoidance behavior. Political Studies Review 15(2): 243–254.

[bibr22-09520767221141121] Hoey M (2001) Textual Interaction: An Introduction to Written Discourse Analysis. London: Routledge.

[bibr23-09520767221141121] Hustedt T Salomonsen HH (2014) Ensuring political responsiveness: politicization mechanisms in ministerial bureaucracies. International Review of Administrative Sciences 80(4): 746–765.

[bibr24-09520767221141121] Jenkins P (2009) Failure to launch: why do some social issues fail to detonate moral panics? The British Journal of Criminology 49(1): 35–47.

[bibr25-09520767221141121] Jenkins L (2011) The difference genealogy makes. Political Studies 59(1): 156–174.

[bibr26-09520767221141121] Kim PS (2021) South Korea’s fast response to coronavirus disease: implications on public policy and public management theory. Public Management Review 23(12): 1736–1747.

[bibr27-09520767221141121] Kissas A (2020) Performative and ideological populism: The case of charismatic leaders on Twitter. Discourse & Society 31(3): 268–284.

[bibr28-09520767221141121] Koon AD Hawkins B Mayhew SH (2016) Framing and the health policy process: a scoping review. Health Policy and Planning 31(6): 801–816. 26873903 10.1093/heapol/czv128 PMC4916318

[bibr29-09520767221141121] Krzyżanowski M Triandafyllidou A Wodak R (2018) The Mediatization and the Politicization of the “Refugee Crisis” in Europe. Journal of Immigrant & Refugee Studies 16(1–2): 1–14.

[bibr30-09520767221141121] Laclau E (2004) Populism: What’s in a name? (eds), Empire & Terror: nationalism/Postnationalism in the New Millennium. Reno: Center for Basque Studies, pp. 103–114.

[bibr31-09520767221141121] Laclau E (2005) On Populist Reason. London: Verso.

[bibr32-09520767221141121] Laclau E Mouffe C (1985) Hegemony and Socialist Strategy. London: Verso.

[bibr33-09520767221141121] Lasco G (2020) Medical populism and the COVID-19 pandemic. Global Public Health 15(10): 1417–1429. 32780635 10.1080/17441692.2020.1807581

[bibr34-09520767221141121] Lasco G Curato N (2019) Medical populism. Social Science & Medicine 221(1): 1–8. 30553118 10.1016/j.socscimed.2018.12.006

[bibr35-09520767221141121] Lasco G Larson HJ (2020) Medical populism and immunisation programmes: Illustrative examples and consequences for public health. Global Public Health 15(3): 334–344. 31630625 10.1080/17441692.2019.1680724

[bibr36-09520767221141121] Massuda A Hone T Leles FAG, et al. (2018) The Brazilian health system at crossroads: progress, crisis and resilience. BMJ Global Health 3: 1–8. 10.1136/bmjgh-2018-000829 PMC6035510 29997906

[bibr37-09520767221141121] Mellan TA Hoeltgebaum H Mishra S, et al. (2020) Report 21: Estimating COVID-19 cases and reproduction number in Brazil. Available at: https://www.imperial.ac.uk/mrc-global-infectious-disease-analysis/covid-19/report-21-brazil/ (accessed 20 May 2021).

[bibr38-09520767221141121] Meyer M (2001) Between theory, method, and politics: positioning of the approaches to CDA. In: Wodak R Meyer M (eds), Methods of Critical Discourse Analysis. London: Sage, pp. 14–32.

[bibr39-09520767221141121] Moffitt B Tormey S (2014) Rethinking Populism: Politics, Mediatisation and Political Style. Political Studies 62(2): 381–397.

[bibr40-09520767221141121] Mudde C (2010) The Populist Radical Right: A Pathological Normalcy. West European Politics 33(6): 1167–1186.

[bibr41-09520767221141121] Müller J-W (2017) What Is Populism? Philadelphia: University of Pennsylvania Press.

[bibr42-09520767221141121] Ongaro E Gong T Jing Y (2018) Toward Multi-Level Governance in China? Coping with complex public affairs across jurisdictions and organizations. Public Policy and Administration 34(2): 105–120.

[bibr43-09520767221141121] Osuna JJO (2021) From chasing populists to deconstructing populism: A new multidimensional approach to understanding and comparing populism. European Journal of Political Research 60(4): 829–853.

[bibr44-09520767221141121] Peters GB (2013) Politicization: What is it and why should we care? In: Neuhold C Vanhoonacker S Verhey L (eds), Civil Servants and Politics: A Delicate Balance. New York: Palgrave Macmillan, pp. 12–24.

[bibr45-09520767221141121] Peters GB Pierre J (2004) Politicization of the Civil Service in Comparative Perspective. New York: Routledge.

[bibr46-09520767221141121] Ramiro L Gomez R (2017) Radical-Left Populism during the Great Recession: Podemos and Its Competition with the Established Radical Left. Political Studies 65(IS): 108–126.

[bibr47-09520767221141121] Reisigl M Wodak R (2009) The discourse-historical approach (DHA). In: Wodak R Meyer M (eds), Methods of Critical Discourse Analysis. 2nd ed. London: Sage, pp. 87–121.

[bibr48-09520767221141121] Scollon R (2001) Action and text: towards an integrated understanding of the place of text in social (inter)action, mediated discourse analysis and the problem of social action. In: Wodak R Meyer M (eds), Methods of Critical Discourse Analysis. London: Sage, pp. 139–195.

[bibr49-09520767221141121] Shaw SE (2010) Reaching the parts that other theories and methods can’t reach: How and why a policy-as-discourse approach can inform health-related policy. Health 14(2): 196–212. 20164166 10.1177/1363459309353295

[bibr50-09520767221141121] Speed E Mannion R (2017) The Rise of Post-truth Populism in Pluralist Liberal Democracies: Challenges for Health Policy. International Journal of Health Policy and Management 6(5): 249–251. 28812811 10.15171/ijhpm.2017.19 PMC5417145

[bibr51-09520767221141121] Speed E Mannion R (2020) Populism and health policy: three international case studies of right-wing populist policy frames. Sociology of Health & Illness 42(8): 1967–1981. 32780437 10.1111/1467-9566.13173

[bibr52-09520767221141121] Titscher S Meyar M Wodak R, et al (2000) Methods of Text and Discourse Analysis. London: Sage.

[bibr53-09520767221141121] Toulmin SE (2003) The Uses of Argument. New York: Cambridge University Press.

[bibr54-09520767221141121] Triandafyllidou A Fotiou A (1998) Sustainability and Modernity in the European Union: A Frame Theory Approach to Policy-Making. Sociological Research Online 3(1): 60–75.

[bibr55-09520767221141121] Ungar S (2001) Moral panic versus the risk society: the implications of the changing sites of social anxiety. The British Journal of Sociology 52(2): 271–291. 11440057 10.1080/00071310120044980

[bibr56-09520767221141121] Van Dijk TA (1998) Ideology: A Multidisciplinary Approach. London: SAGE.

[bibr57-09520767221141121] Van Dijk TA (2003) Political Discourse and Ideology. Doxa Comunicacion 1: 207–225.

[bibr58-09520767221141121] Van Dijk TA (2006) Politics, Ideology, and Discourse. In: Brown K (ed), Encyclopedia of Language & Linguistics. Oxford: Elsevier Pergamon, pp. 728–740.

[bibr59-09520767221141121] Van Leeuwen T Wodak R (1999) Legitimizing immigration control: A discourse historical analysis. Discourse Studies 1(1): 83–118.

[bibr60-09520767221141121] Weyland K (2001) Clarifying a Contested Concept Populism in the Study of Latin American Politics. Comparative Politics 34(1): 1–22.

[bibr61-09520767221141121] Widdowson HG (1995) Discourse analysis: a critical review. Language and Literature 4(3): 157–172.

[bibr62-09520767221141121] Wodak R (2001) What CDA is about – a summary of its history, important concepts and its developments. In: Wodak R Meyer M (eds), Methods of Critical Discourse Analysis. London: Sage, pp. 1–13.

[bibr63-09520767221141121] Wodak R Meyer M (2009) Critical Discourse Analysis: History, Agenda, Theory, and Methodology. In: Wodak R Meyer M (eds), Methods of Critical Discourse Analysis. 2nd ed. London: Sage, pp. 1–33.

[bibr64-09520767221141121] Wondreys J Mudde C (2020) Victims of the Pandemic? European Far-Right Parties and Covid-19. Nationalities Papers 1–18.

[bibr65-09520767221141121] Wood M (2016) Politicisation, Depoliticisation and Anti-Politics: Towards a Multilevel Research Agenda. Political Studies Review 14(4): 52–533.

