# Supplementary material for: ‘This is just a little flu’: analysing medical populist discourses on the Covid-19 pandemic in Brazil
Source: Public Policy Adm. 2022 Nov 30:09520767221141121. doi: 10.1177/09520767221141121 (PMC9720056; doi:10.1177/09520767221141121)

Supplemental Material

Appendix A

Discursive sources, documents and political actors included in analysis

Specific political units

Federal, state and municipal governments

Ministry of Health official website
https://www.saude.gov.br/noticias/agencia-saude/46412-brasil-apresenta-acoes-para-enfrentamento-do-novo-coronavirus

House of Representatives official website
https://www.camara.leg.br/noticias/648863-camara-aprova-auxilio-de-r-600-para-pessoas-de-baixa-renda-durante-epidemia

Senate official website
https://www12.senado.leg.br/noticias/materias/2020/04/29/ministro-da-saude-enfatiza-importancia-de-201cregionalizar201d-combate-ao-coronavirus

National and International Response to Fight the New Coronavirus – Ministry of Health https://coronavirus.saude.gov.br/linha-do-tempo/#dez2019

Supreme Court Justice

Specific social, political and scientific actors:

President of Brazil

Vice President of Brazil

Chief Minister of the Civil House

Minister of Health 1 – Jan2019 – Apr2020

Minister of Health 2 – Apr2020 – May2020

Secretary of Science, Technology and Strategic Supplies of the Ministry of Health

Minister of Economy

Minister of Justice

Minister of Defence

Minister of Citizenship

Minister of Women, Family, and Human Rights

Governor of the State of São Paulo

Governor of the State of Rio de Janeiro

Governor of the State of Espírito Santo

Governor of the State of Goiás

Governor of the State of Santa Catarina

Governor of the State of Pará

Director of the Department of Immunization and Transmissible Diseases of the Ministry of Health

Chair of the National Council of State Health Secretaries (CONASS)

Coordinator of State Health and Disease Control Secretary of São Paulo

State Health Secretary of São Paulo

Coordinator of the Coronavirus Contingency Center of São Paulo

President of the House of Representatives

President of Senate

Director of the National Health Surveillance Agency (ANVISA)

Executive Secretary of the Ministry of Health 1 – Jan2019 – Apr2020

Executive Secretary of the Ministry of Health 2 – Apr2020 – Jun2020

Secretary of Health Surveillance

Mayor of Manaus

WHO Director-General

Specific semiotic media and genres

Special Epidemiological Bulletin 19 – Department of Epidemiological Surveillance (SVS)

Epidemiological Bulletin COE 1/2020 – Department of Epidemiological Surveillance (SVS)

National Response Plan to Public Health Emergency – Ministry of Health

Covid-19 Management Strategy – Ministry of Health

CFM Report n. 4/2020 – Brazilian Federal Council of Medicine (CFM)

Technical Note GVIMS/GGTES/ANVISA n. 04/2020 – National Health Surveillance Agency (ANVISA)

Measures Adopted by the Federal Government to Combat the Coronavirus – Presidency of the Republic
https://www.gov.br/casacivil/pt-br/assuntos/noticias/2020/maio/medidas-adotadas-pelo-governo-federal-no-combate-ao-coronavirus-8-de-maio

Law n. 13979/2020
http://www.planalto.gov.br/ccivil_03/_ato2019-2022/2020/lei/l13979.htm

Provisional Measure n. 926/2020
https://www.congressonacional.leg.br/materias/ medidas-provisorias/-/mpv/141144

Decree n. 10211/2020
http://www.planalto.gov.br/ccivil_03/_ato2019-2022/2020/decreto/D10211.htm

Ordinance n. 356/2020
https://www.in.gov.br/en/web/dou/-/portaria-n-356-de-11-de-marco-de-2020-247538346

Ordinance n. 126/2020
https://www.in.gov.br/en/web/dou/-/portaria-n-126-de-19-de-marco-de-2020-248881688

Interministerial Ordinance n. 5/2020
https://www.in.gov.br/en/web/dou/-/portaria-interministerial-n-5-de-17-de-marco-de-2020-248410549

Interministerial Ordinance n. 203/2020
https://www.in.gov.br/en/web/dou/-/portaria-interministerial-n-203-de-28-de-abril-de-2020-254282950

Lawsuit against Noncompliance with Fundamental Precept (ADPF) n. 672/2020 – Brazilian Supreme Court

Letter from Governors to the Federal Government – COSUD

World Health Organization Covid-19 Virtual Press Conference
https://www.who.int/docs/default-source/coronaviruse/transcripts/who-audio-emergencies-coronavirus-press-conference-full-30mar2020.pdf?sfvrsn=6b68bc4a_2

Agência Brasil government news agency

G1.Globo news

R7 news

Jornal o Globo news

Band Jornalismo news

Estadão news

IstoÉ Dinheiro news

Época Negócios news

GZH Política News

Exame news

Folha de S.Paulo news

Folha de Pernambuco news

UOL news

BBC Brasil news

Estado de Minas news

Veja news

Correio Braziliense news

Valor Econômico news

O Antagonista newsletter

Aos Fatos news check

Catraca Livre newsletter

Poder 360 newsletter

YouTube Live
https://www.youtube.com/watch?v=MaWEBJBztYw&ab_channel=BolsonaroTV

YouTube Live
https://www.youtube.com/watch?v=ZLIUvoZDSFc&ab_channel=JairBolsonaro

YouTube Live
https://www.youtube.com/watch?v=hH0JhakIwf0&ab_channel=JairBolsonaro

YouTube Channel
https://www.youtube.com/watch?v=VTCHQkJqv3I&ab_channel=DWBrasil

YouTube Channel
https://www.youtube.com/watch?v=q0T07fGritE&ab_channel=Estad%C3%A3o

YouTube Channel
https://www.youtube.com/watch?v=QKhqOngkV6M&ab_channel=FlavioBolsonaro

YouTube Channel
https://www.youtube.com/watch?v=F9jXlF2ExQE&ab_channel=JairBolsonaro

YouTube Channel
https://www.youtube.com/watch?v=wPQX_GHsSr8&ab_channel=GazetadoPovo

YouTube Official Speech

Globo TV<br>SBT TV<br>Record TV<br>Rede TV<br>CNN Brasil TV<br>NSC TV<br>Jovem Pan Radio<br>Rede Brasil Atual Radio<br>REUTERS news agency<br>El Pais news<br>Euronews News<br>BBC news<br>The Guardian news<br>DW News<br>France24 news	https://www.youtube.com/watch?v=bS2qiXHtMnI&ab_channel=TVBrasilGov<br>YouTube Official Speech<br>https://www.youtube.com/watch?v=fnJov5K1BSw&ab_channel=Planalto<br>YouTube Official Speech<br>https://www.youtube.com/watch?v=16RR2rG_AKA&ab_channel=Planalto<br>YouTube Press Conference<br>https://www.youtube.com/watch?v=42nTUhviteg&ab_channel=TVBrasilGov<br>YouTube Press Conference<br>https://www.youtube.com/watch?v=lhltNqinvm4&ab_channel=TVBrasilGov<br>LinkedIn Article<br>https://www.linkedin.com/pulse/covid-19-como-conduzir-o-sistema-de-sa%C3%BAde-e-brasil-nelson-teich/<br>Twitter Posts

List of Open and Axial Coding

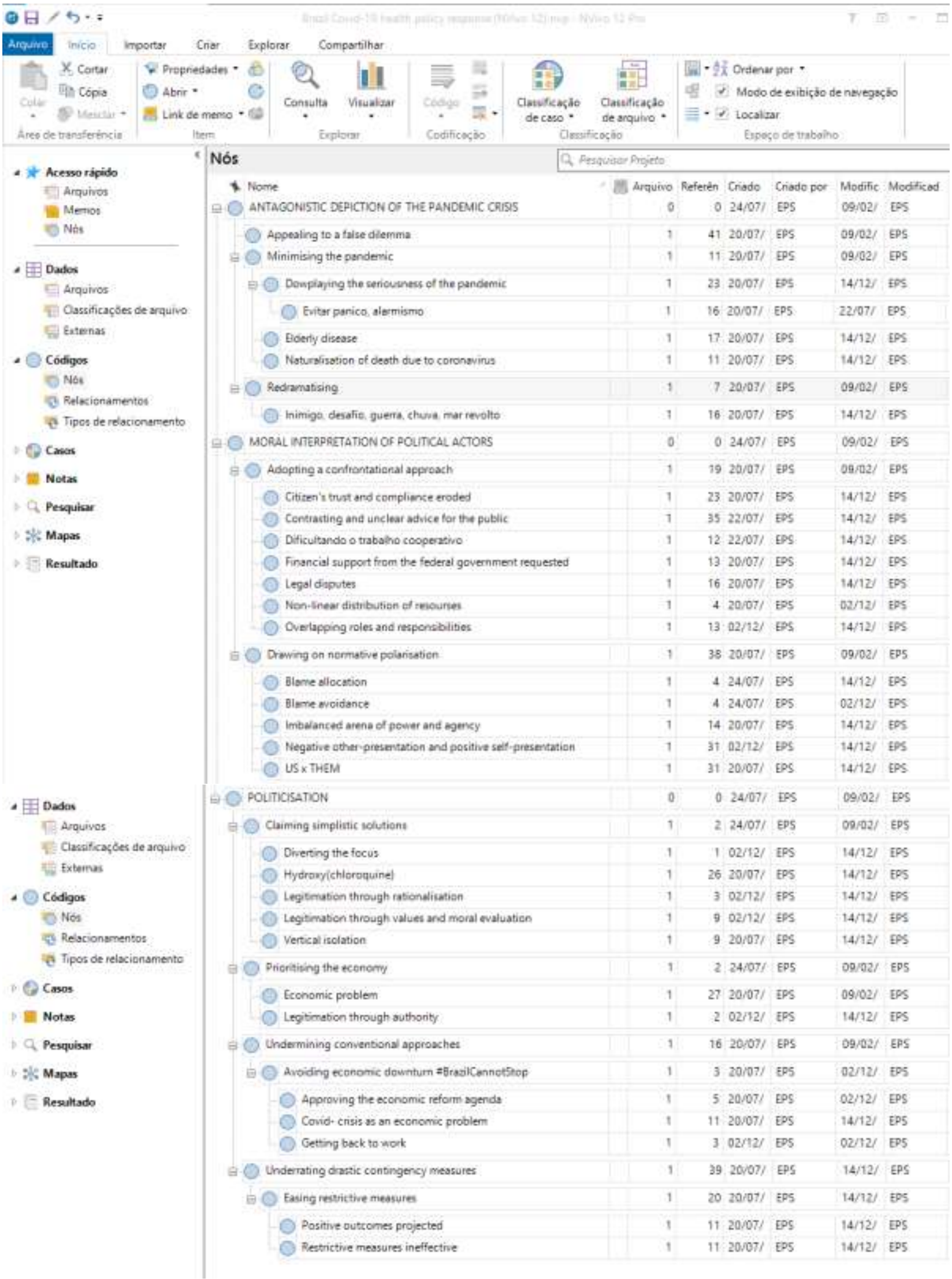

Nome	Arquivo	Referên	Criado	Criado por	Modific	Modificad
ANTAGONISTIC DEPICTION OF THE PANDEMIC CRISIS	0	0	24/07/	EPS	09/02/	EPS
Appealing to a false dilemma	1	41	20/07/	EPS	09/02/	EPS
Minimising the pandemic	1	11	20/07/	EPS	09/02/	EPS
Dowplaying the seriousness of the pandemic	1	23	20/07/	EPS	14/12/	EPS
Evitar panico, alermismo	1	16	20/07/	EPS	22/07/	EPS
Elderly disease	1	17	20/07/	EPS	14/12/	EPS
Naturalisation of death due to coronavirus	1	11	20/07/	EPS	14/12/	EPS
Redramatising	1	7	20/07/	EPS	09/02/	EPS
Inimigo, desafio, guerra, chuva, mar revolto	1	16	20/07/	EPS	14/12/	EPS
MORAL INTERPRETATION OF POLITICAL ACTORS	0	0	24/07/	EPS	09/02/	EPS
Adopting a confrontational approach	1	19	20/07/	EPS	08/02/	EPS
Citizen's trust and compliance eroded	1	23	20/07/	EPS	14/12/	EPS
Contrasting and unclear advice for the public	1	35	22/07/	EPS	14/12/	EPS
Dificultando o trabalho cooperativo	1	12	22/07/	EPS	14/12/	EPS
Financial support from the federal government requested	1	13	20/07/	EPS	14/12/	EPS
Legal disputes	1	16	20/07/	EPS	14/12/	EPS
Non-linear distribution of resourses	1	4	20/07/	EPS	02/12/	EPS
Overlapping roles and responsibilities	1	13	02/12/	EPS	14/12/	EPS
Drawing on normative polarisation	1	38	20/07/	EPS	09/02/	EPS
Blame allocation	1	4	24/07/	EPS	14/12/	EPS
Blame avoidance	1	4	24/07/	EPS	02/12/	EPS
Imbalanced arena of power and agency	1	14	20/07/	EPS	14/12/	EPS
Negative other-presentation and positive self-presentation	1	31	02/12/	EPS	14/12/	EPS
US x THEM	1	31	20/07/	EPS	14/12/	EPS
POLITICISATION	0	0	24/07/	EPS	09/02/	EPS
Claiming simplistic solutions	1	2	24/07/	EPS	09/02/	EPS
Diverting the focus	1	1	02/12/	EPS	14/12/	EPS
Hydroxy(chloroquine)	1	26	20/07/	EPS	14/12/	EPS
Legitimation through rationalisation	1	3	02/12/	EPS	14/12/	EPS
Legitimation through values and moral evaluation	1	9	02/12/	EPS	14/12/	EPS
Vertical isolation	1	9	20/07/	EPS	14/12/	EPS
Prioritising the economy	1	2	24/07/	EPS	09/02/	EPS
Economic problem	1	27	20/07/	EPS	09/02/	EPS
Legitimation through authority	1	2	02/12/	EPS	14/12/	EPS
Undermining conventional approaches	1	16	20/07/	EPS	09/02/	EPS
Avoiding economic downturn #BrazilCannotStop	1	3	20/07/	EPS	02/12/	EPS
Approving the economic reform agenda	1	5	20/07/	EPS	02/12/	EPS
Covid- crisis as an economic problem	1	11	20/07/	EPS	14/12/	EPS
Getting back to work	1	3	02/12/	EPS	02/12/	EPS
Underrating drastic contingency measures	1	39	20/07/	EPS	14/12/	EPS
Easing restrictive measures	1	20	20/07/	EPS	14/12/	EPS
Positive outcomes projected	1	11	20/07/	EPS	14/12/	EPS
Restrictive measures ineffective	1	11	20/07/	EPS	14/12/	EPS

Example of Discursive Corpus

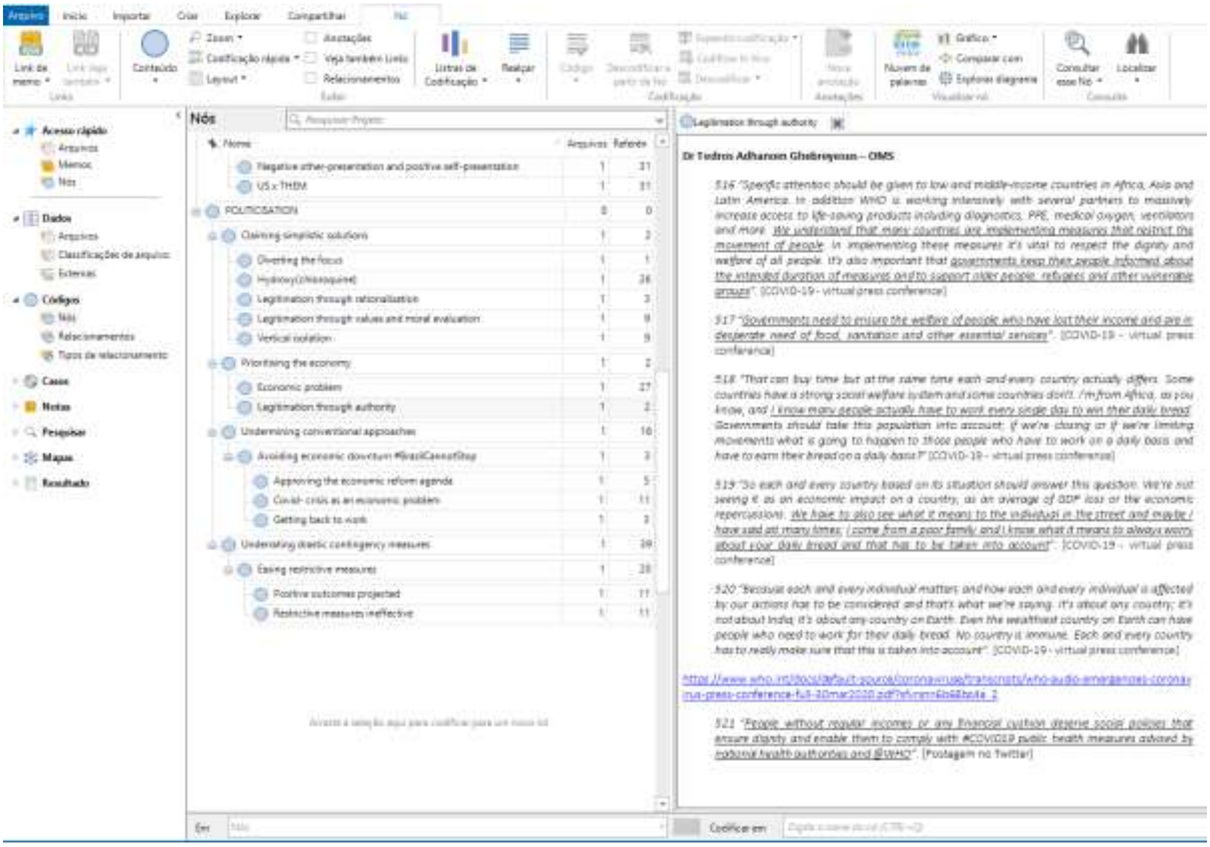

Supplement: Supplemental Material - ‘This is just a little flu’: analysing medical populist discourses on the Covid-19 pandemic in Brazil [file sj-pdf-1-ppa-10.1177_09520767221141121.pdf]
